# Antimicrobial peptides trigger a division block in *Escherichia coli* through stimulation of a signalling system

**DOI:** 10.1038/ncomms12340

**Published:** 2016-07-29

**Authors:** Srujana S. Yadavalli, Jeffrey N. Carey, Rachel S. Leibman, Annie I. Chen, Andrew M. Stern, Manuela Roggiani, Andrew M. Lippa, Mark Goulian

**Affiliations:** 1 Department of Biology, School of Arts and Sciences, University of Pennsylvania, Philadelphia, Pennsylvania 19104, USA; 2 Department of Biochemistry and Molecular Biophysics, Perelman School of Medicine, University of Pennsylvania, Philadelphia, Pennsylvania 19104, USA; 3 Department of Microbiology, Perelman School of Medicine, University of Pennsylvania, Philadelphia, Pennsylvania 19104, USA; 4 Department of Physics, School of Arts and Sciences, University of Pennsylvania, Philadelphia, Pennsylvania 19104, USA

## Abstract

Antimicrobial peptides are an important component of the molecular arsenal employed by hosts against bacteria. Many bacteria in turn possess pathways that provide protection against these compounds. In *Escherichia coli* and related bacteria, the PhoQ/PhoP signalling system is a key regulator of this antimicrobial peptide defence. Here we show that treating *E. coli* with sublethal concentrations of antimicrobial peptides causes cells to filament, and that this division block is controlled by the PhoQ/PhoP system. The filamentation results from increased expression of QueE, an enzyme that is part of a tRNA modification pathway but that, as we show here, also affects cell division. We also find that a functional YFP–QueE fusion localizes to the division septum in filamentous cells, suggesting QueE blocks septation through interaction with the divisome. Regulation of septation by PhoQ/PhoP may protect cells from antimicrobial peptide-induced stress or other conditions associated with high-level stimulation of this signalling system.

## Abstract (web summary)

The PhoQ/PhoP system regulates antimicrobial peptide defense in bacteria. Here the authors show that at sublethal concentrations of antimicrobial peptides, PhoPQ induces QueE, that then localizes to the divisome and blocks cell division independently of its function in queuosine biosynthesis.

## Graphical abstract

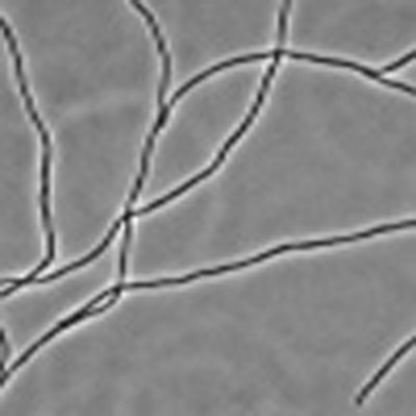

Antimicrobial peptides are widely produced by animals, plants and many other organisms as a defence against bacteria. These compounds span a diverse range of molecular species, but many consist of amphipathic cationic molecules that are able to transit and/or disrupt the bacterial cell envelope. Not surprisingly, bacteria have evolved the ability to detect and mount a defence against these compounds. In *Escherichia coli*, Salmonella and many related bacteria, one of the major pathways involved in sensing and regulating resistance to cationic antimicrobial peptides is controlled by the PhoQ/PhoP two-component system^1^,^2^,^3^. The sensor kinase PhoQ detects the presence of these peptides, as well as low concentrations of Mg^2+^ and Ca^2+^, and controls the phosphorylation state of the response regulator PhoP, which functions as a transcription factor^2^,^3^,^4^,^5^,^6^. Some PhoP-regulated genes encode proteins that clearly provide protection from antimicrobial peptides or the stresses associated with other PhoQ-stimulating conditions. For example, the PhoP-regulated genes *pagP*, *mgtA* and *hdeA* encode an enzyme that confers resistance to antimicrobial peptides, a high-affinity magnesium transporter, and a chaperone that protects against acid stress, respectively^7^,^8^,^9^,^10^,^11^. For many other genes in the PhoP regulon, on the other hand, their expression might not provide protection against damage from the PhoQ stimulus but instead confer a selective advantage for surviving other aspects of the environment that are strongly correlated with PhoQ activation.

The stimuli that activate PhoQ are found in different settings and exert complex effects on the bacterial cell, which likely accounts at least in part for the diverse functions of proteins regulated by PhoP. In light of this diversity, there is no reason to expect PhoP regulon members to be activated to similar extents for all PhoQ stimulating conditions. Indeed, the expression of some PhoP-regulated genes depends both on the extent of PhoP phosphorylation, which depends on the magnitude and type of stimulus, as well as the action of additional regulators, to provide multiple levels of control^12^,^13^,^14^. As part of this regulation, feedback loops act within the PhoQ/PhoP circuit to modulate phosphorylated PhoP (PhoP-P) levels^14^,^15^,^16^,^17^. For example, activation of *phoPphoQ* transcription by PhoP-P (autoregulation)^15^ extends the output range of the system at high stimulus^18^. Another example is negative feedback from the PhoP-regulated protein MgrB^17^,^19^, which has the effect of tempering PhoQ activity and extending the dynamic range of input signals. Inactivation of *mgrB* has been shown to be one of the primary pathways for acquired resistance to colistin (an antimicrobial peptide) among clinical isolates of *Klebsiella pneumoniae*^20^,^21^.

Here we show that high-level activation of PhoQ from sublethal doses of cationic antimicrobial peptides inhibits cell division, causing *E. coli* cells to grow as long filaments. Similar behaviour can also be achieved for other conditions that strongly activate PhoQ, such as growth of a Δ*mgrB* strain in low Mg^2+^. The filamentous cells have a continuous cytoplasm and intact FtsZ rings, suggesting a block downstream of Z-ring formation in the cell division pathway. From a suppressor screen, we determined that filamentation depends on QueE, an enzyme involved in the biosynthesis of a hyper-modified guanosine (queuosine) found in certain tRNAs^22^,^23^. We further find that PhoP regulates *queE* transcription, and that increased expression of QueE inhibits cell division. However, QueE's effect on cell division is independent of the queuosine biosynthesis pathway. Imaging of cells expressing a functional YFP-QueE fusion indicates that under filamenting conditions QueE localizes to the divisome, suggesting that the protein interacts directly with division machinery.

## Results

### Antimicrobial peptides trigger PhoQ-dependent filamentation

**Figure 1:**
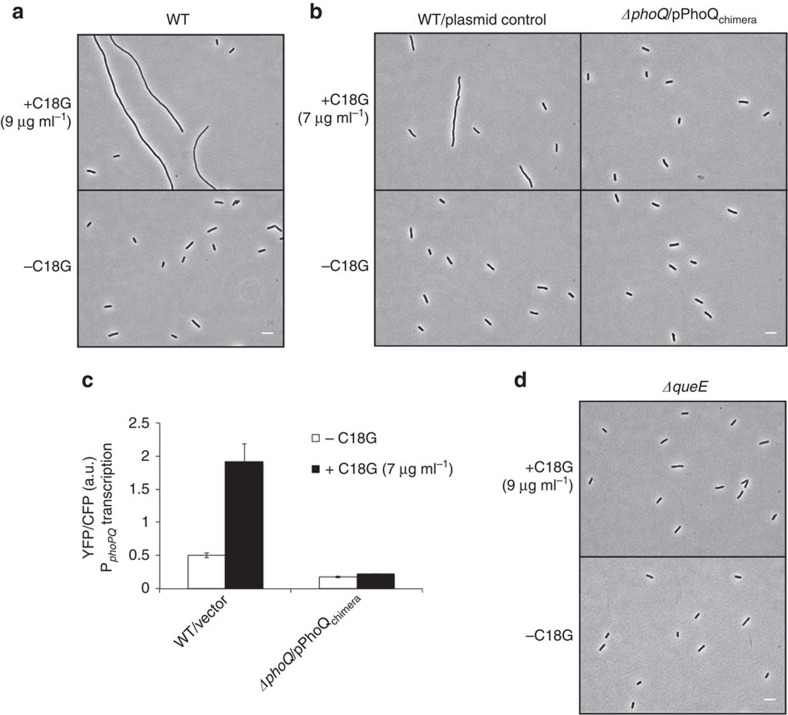
Antimicrobial peptide stimulation of the *E. coli* PhoQ/PhoP system causes cells to filament. (**a**) Phase-contrast micrographs of wild-type (MG1655) cells grown in the presence or absence of C18G. (**b**) Phase-contrast micrographs of wild-type cells containing control vector (TIM148/pGB2) and *ΔphoQ* cells containing a plasmid encoding PhoQ_chimera_ (TIM229/pLPQ*2) grown in the presence or absence of C18G. (**c**) Measurement of *phoPQ* promoter activity (which is regulated by PhoP-P) from a P_*phoPQ*_-*yfp* transcriptional reporter in wild-type cells containing control vector (TIM148/pGB2) and *ΔphoQ* cells containing a plasmid encoding PhoQ_chimera_ (TIM229/pLPQ*2) grown in the presence or absence of C18G. PhoQ_chimera_, which is not stimulated by C18G but has a basal level of PhoQ activity, was used to avoid the high toxicity of C18G in *ΔphoQ* strains. (See text for details.) Data represent means and s.d.'s from at least three independent experiments. (**d**) Phase-contrast micrographs of *ΔqueE* cells (SAM96) grown in the presence or absence of C18G. Cells were grown in minimal medium containing 0.1 mM Mg^2+^and the indicated concentration of C18G to an OD_600_=0.2–0.3 and imaged by microscopy. Scale bar, 5 μm.

Wild-type *E. coli* grown in the presence of sub-lethal levels of the antimicrobial peptide C18G formed long filaments ranging from tens to hundreds of microns in length and with considerable heterogeneity in size (Fig. 1a, see Supplementary Table 1 for average cell length measurements). To determine if this filamentation is PhoQ-dependent, we attempted to examine the cellular morphology of *ΔphoQ* cells treated with C18G. However, we found that C18G concentrations that cause wild-type cells to filament prevented growth of *ΔphoQ* cells. This increased toxicity for cells lacking PhoQ is unsurprising as the PhoQ/PhoP network plays a crucial role in defence against cationic antimicrobial peptides^1^,^2^,^3^. As an alternative approach, we used a chimeric PhoQ (PhoQ_chimera_) consisting of the cytoplasmic and transmembrane domains from *E. coli* PhoQ and the periplasmic domain from *Pseudomonas aeruginosa* PhoQ^24^. This chimera has partial PhoQ kinase activity^17^,^24^, which we reasoned might provide sufficient PhoP-regulated gene expression to afford protection against antimicrobial peptides. A strain containing a deletion of the chromosomal copy of *phoQ* and carrying *phoQ*_chimera_ on a plasmid grew without forming filaments when treated with 7 μg ml^−1^ C18G. However, wild-type cells containing a control vector formed filaments under the same growth conditions (Fig. 1b). (We used a lower concentration of C18G in these experiments compared with those used for the experiments shown in Fig. 1a because cells containing *phoQ*_chimera_ were more sensitive than wild-type cells to C18G.)

To infer PhoQ/PhoP activity, we used a PhoP-regulated promoter driving transcription of *yfp*, the gene for yellow fluorescent protein^12^. Whereas treatment with C18G showed an increase in fluorescence for the reporter strain containing wild-type *phoQ*, consistent with the activation of the PhoQ/PhoP system, there was no significant change for the reporter strain expressing PhoQ_chimera_, indicating that the chimeric protein is insensitive to stimulation with C18G (Fig. 1c).

**Figure 2:**
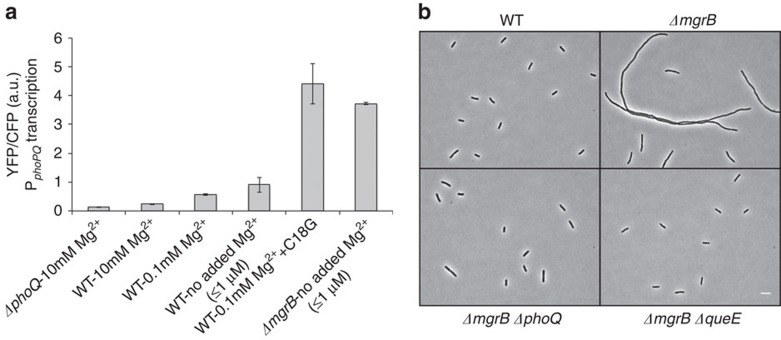
Filamentation is associated with conditions that strongly stimulate PhoQ/PhoP. (**a**) Measurement of *phoPQ* promoter activity from a P_*phoPQ*_-*yfp* transcriptional reporter in wild-type (TIM148), *ΔphoQ* (TIM229) and *ΔmgrB* (AML22) strains in minimal medium at indicated Mg^2+^ concentration. For wild-type cells grown in the presence of antimicrobial peptide C18G, C18G was added to a final concentration of 9 μg ml^−1^. Cells were grown until an OD_600_=0.2–0.3 and fluorescence was measured by microscopy. Data represent means and s.d.'s from at least three independent experiments. (**b**) Cellular morphology of wild-type (TIM92), *ΔmgrB* (AML20), *ΔmgrB ΔphoQ* (AML17) and *ΔmgrB ΔqueE* (JNC21) cells grown in minimal medium with no added Mg^2+^. Scale bar, 5 μm.

Comparison of PhoQ/PhoP-regulated transcription for growth in different concentrations of Mg^2+^ or treatment with C18G (at concentrations that cause cells to filament) indicated that the latter condition leads to significantly higher levels of activation of the PhoQ/PhoP system, with an increase of 3.5–4-fold compared with wild-type cells grown in medium with no added Mg^2+^ (≤1 μM; Fig. 2a). Thus particularly high levels of PhoQ/PhoP stimulation can be reached with sublethal concentrations of antimicrobial peptides.

### MgrB-null strains filament at low Mg^2+^

We found that wild-type cells did not filament when grown in medium with no added Mg^2+^ (Fig. 2b). PhoQ is stimulated under these growth conditions but, as noted above, the level of stimulation is not as high as the level reached with antimicrobial peptide treatment (Fig. 2a). PhoQ stimulation is further increased, however, in cells that lack MgrB, a negative regulator of PhoQ (Fig. 2a). When we examined the morphology of *ΔmgrB* cells growing in low-Mg^2+^ medium we found that the cells grew as long filaments (Fig. 2b, Supplementary Table 1). We were able to detect filament formation for a range of magnesium concentrations, with the extent of filamentation greatest in medium with no added Mg^2+^ (≤1 μM) and no detectable filamentation for Mg^2+^⩾1 mM (Supplementary Fig. 1). Moreover, following addition of 1 mM Mg^2+^, filamentous cells septate and continue to grow as smaller cells. We also confirmed that MgrB expressed from a plasmid complements the *mgrB* deletion, indicating that filamentation is due to the absence of this small membrane protein (Supplementary Fig. 2). In addition, *ΔmgrB ΔphoQ* cells grown in low Mg^2+^ do not filament, indicating that the phenotype is PhoQ-dependent (Fig. 2b).

Taken together, the above results for C18G treatment of wild-type cells and for *ΔmgrB* cells in low Mg^2+^ indicate that strong stimulation of the *E. coli* PhoQ/PhoP two-component system leads to filament formation. We also tested two other ways to strongly stimulate the PhoQ/PhoP system: expression of a phosphatase-deficient PhoQ^18^ and expression of SafA, a small protein that stimulates PhoQ^25^. Both resulted in cell filamentation (Supplementary Fig. 3), further confirming that the phenotype depends only on strong stimulation of the PhoQ/PhoP system and is not specific to cationic antimicrobial peptide or low Mg^2+^ stress. For further characterization of cell filaments and the filamentation mechanism, described below, we took advantage of the filamentation of *ΔmgrB* cells to avoid complications from the toxicity (and high cost) of antimicrobial peptides.

### Filaments have a continuous cytoplasm and intact FtsZ rings

Filamentous cells stained with the membrane dye FM4–64 displayed smooth contours with no visible septa (Supplementary Fig. 4a). In addition, fluorescence recovery after photobleaching (FRAP) experiments showed a rapid recovery to uniform fluorescence (Supplementary Fig. 4b). These results indicate that filaments have a continuous cytoplasm and therefore the block in cell division is due to inhibition of septation.

**Figure 3:**
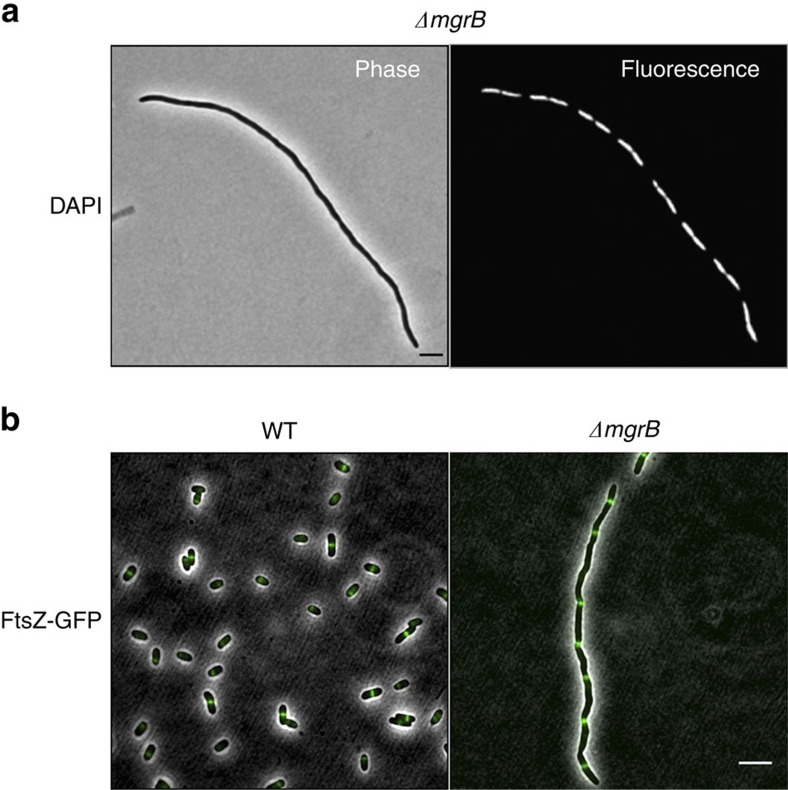
Chromosomal segregation and FtsZ ring formation are not impaired in filamenting cells. (**a**) Phase contrast and fluorescence micrographs of *ΔmgrB* (AML20) cells stained with DAPI. (**b**) Fluorescence micrographs of wild-type *E. coli* (EC448) and *ΔmgrB* (JNC19) cells showing FtsZ-GFP localization. All cultures were grown in minimal medium with no added Mg^2+^. Scale bar, 5 μm.

To gain further insight into the mechanism of filamentation induced by activation of PhoQ/PhoP, we tested pathways associated with inhibiting septation. Staining with DAPI and visualization by fluorescence microscopy revealed that cell filaments contain multiple nucleoids, evenly distributed along the length of the cell (Fig. 3a). Furthermore, deletion of *sulA*, which is induced by the SOS response and encodes an inhibitor of FtsZ ring formation^26^,^27^, did not affect filament formation (Supplementary Fig. 5). These results suggest that the filaments are not a result of DNA damage. In addition, imaging of an FtsZ–GFP protein fusion in *ΔmgrB* cells shows a striped pattern of FtsZ rings (Fig. 3b), indicating that the block in septation is downstream of FtsZ-ring formation. The mean distance between Z-rings in filamenting cells was 5.9 μm (*n*=61; s.d. 1.8 μm). Similar spacing between Z-rings has been reported previously for several other examples of cell filamentation associated with perturbation of cell division proteins^28^,^29^.

### Deletion of the gene *queE* suppresses filamentation

To identify genes that participate in this PhoQ/PhoP-regulated filamentation pathway, we developed a method to screen for filamentation suppressors on solid media. Colonies of a *ΔmgrB* strain grown on low-Mg^2+^ minimal medium solidified with agarose were composed of highly filamentous cells. Surprisingly, these colonies had a similar size and morphology to colonies of wild-type cells growing on most agarose preparations. However, for low-Mg^2+^ plates prepared with a specific agarose (4% SeaPlaque), wild-type and *ΔmgrB* strains showed a clear difference in colony morphology (Supplementary Fig. 6a). On this medium, a colony consisting of the non-filamentous wild-type cells appeared smaller, smoother, more circular and more opaque than colonies of the filamentous (*ΔmgrB*) cells. We used this difference in morphology as the basis of a transposon mutagenesis screen for suppressors of filamentation (Supplementary Fig. 6b). From this screen, we isolated a suppressor containing an insertion in the gene *queE*. An in-frame deletion of *queE* in a *ΔmgrB* strain, constructed by P1 transduction of the *queE* deletion in the Keio collection^30^, similarly suppressed filamentation of a *ΔmgrB* strain (Fig. 2b), as well as filamentation induced by treatment with the antimicrobial peptide C18G (Fig. 1d). We also verified that introduction of a plasmid-borne copy of *queE* restored filamentation, establishing that the suppression is specifically due to the loss of *queE* expression (Supplementary Fig. 7).

### Queuosine biosynthesis does not affect filamentation

QueE catalyses the formation of 7-carboxy-7-deazaguanine (CDG), which is an intermediate in the biosynthesis of queuosine, a hyper-modified guanosine that is found in some tRNAs and whose physiological significance is not understood^31^,^32^,^33^,^34^,^35^. Since the deletion of *queE* suppressed the cell division block observed in *ΔmgrB* strains, we asked whether the queuosine biosynthetic pathway was responsible for filamentation. Enzymes QueD, QueE and QueC act sequentially in the formation of the intermediate preQ_0_, which is eventually converted through a series of reactions to queuosine^32^ (Supplementary Fig. 8a). If filamentation suppression in the *ΔqueE ΔmgrB* strain were due to the absence of either CDG or preQ_0_, then deletions of *queD* or *queC* in a *ΔmgrB* strain should display a phenotype similar to that of *ΔqueE* *ΔmgrB*. However, deletion of neither *queD* nor *queC* suppressed filamentation (Supplementary Fig. 8b). In addition, we found that a *queE* deletion suppresses filamentation in a *ΔmgrB ΔqueD* strain. This latter observation rules out the possibility that accumulation of 6-carboxy-5,6,7,8-tetrahydropterin, which is the substrate for QueE and is synthesized by QueD (Supplementary Fig. 8a), is responsible for the suppression of filamentation by *ΔqueE*.

### Increased *queE* expression causes filamentation

Since a *queE* deletion suppresses filamentation, we hypothesized that increased *queE* expression in wild-type cells would have the opposite effect and cause filamentation. Indeed, it was previously reported that overexpression of *queE* (formerly named *ygcF)* causes filamentous biofilm formation^36^. We found that inducing *queE* expression from an IPTG-inducible promoter on a plasmid caused wild-type cells to filament (Supplementary Fig. 9). We also confirmed that filamentation from increased *queE* expression does not require *queD* or *queC*, consistent with the results above.

**Figure 4:**
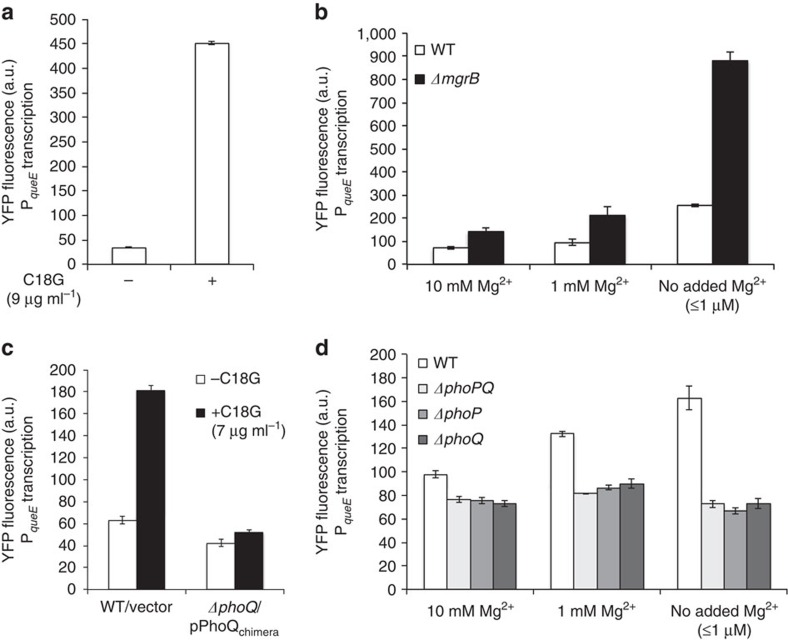
The PhoQ/PhoP system regulates *queE* transcription. (**a**) YFP fluorescence of a *queE-yfp* operon fusion measured in wild-type cells (SAM54) growing in the presence (+) or absence (−) of C18G (9 μg ml^−1^) in minimal medium containing 0.1 mM Mg^2+^. Data represent the means and ranges of two independent cultures. (**b**) YFP fluorescence measured from a *queE-yfp* operon fusion in wild-type (SAM54) and *ΔmgrB* (SAM55) strains in minimal medium at the indicated Mg^2+^ concentrations. Data represent means and s.d.'s from at least three independent experiments. (**c**) YFP fluorescence of a *queE-yfp* operon fusion measured in wild-type cells containing a control plasmid (SAM54/pGB2) and *ΔphoQ* cells containing a plasmid encoding PhoQ_chimera_ (SAM60/pLPQ*2) grown in the presence (+) or absence (−) of C18G (7 μg ml^−1^) in minimal medium containing 0.1 mM Mg^2+^. Data represent the means and s.d.'s from at least three independent experiments. The PhoQ_chimera_ protein, which is not stimulated by C18G but has a basal level of PhoQ activity, was used to avoid problems due to the high toxicity of C18G in *ΔphoQ* strains. (**d**) YFP fluorescence measured from a *queE-yfp* operon fusion in wild-type (SAM54), *ΔphoPQ* (SAM141), *ΔphoP* (SAM142) and *ΔphoQ* (SAM60) strains in minimal medium at the indicated Mg^2+^ concentration. Data represent the means and ranges of two independent cultures.

The fact that QueE-dependent filamentation is associated with high-level activation of the PhoQ/PhoP system (as shown above) led us to hypothesize that *queE* may be regulated by PhoQ/PhoP and that filamentation may be due to increased expression of *queE* when PhoQ is strongly stimulated. To test this possibility, we constructed an operon fusion of *yfp* downstream of *queE* so that the two genes are co-transcribed with separate stop codons and ribosome binding sites. Treatment of wild-type cells with the antimicrobial peptide C18G produced a strong, roughly 10-fold, increase in *queE* transcription (Fig. 4a). Wild-type and *ΔmgrB* strains also showed increases in *queE* transcription in response to low Mg^2+^ (Fig. 4b), with *queE* transcription significantly higher for *ΔmgrB* than for wild type. Measurements with PhoQ-null strains (for different Mg^2+^ concentrations) or with PhoQ_chimera_ (for C18G treatment) indicated that these changes in *queE* transcription are dependent on the PhoQ/PhoP system (Fig. 4c,d).

To test whether PhoP binds near the *queE* promoter, we performed electrophoretic mobility shift assays (EMSAs) with two segments of DNA upstream of *queE*: segment X1, consisting of 300 bp upstream of the *queE* start codon, and segment X2, consisting of a 300 bp segment that overlaps the last three bp of X1 (Supplementary Fig. 10). The choice of the segment X2 was motivated by our observation that deletion of *yqcG*, the gene immediately upstream of *queE*, in a *ΔmgrB* strain led to particularly high levels of *queE* expression and very strong filamentation (Supplementary Fig. 1, Supplementary Table 1). EMSAs showed that phosphorylated PhoP (PhoP-P) binds X1, suggesting that this region is involved in *queE* regulation (Supplementary Fig. 10). In addition, PhoP-P binds X2, indicating the presence of additional PhoP boxes in this region that may contribute to the regulation of *queE* expression.

### Mutations in cell division genes suppress filamentation

To gain further insight into the regulation of cell division by QueE, we screened for spontaneous mutants that suppress filamentation when QueE is highly expressed. We used a *ΔmgrB ΔyqcG* strain (SAM4) that, as noted above, transcribes *queE* at a high level and has a severe filamentation phenotype. This strain forms filaments even at 1 mM Mg^2+^ (Supplementary Fig. 1a) and grows more slowly than the wild-type strain on low-Mg^2+^ plates. We looked for spontaneous mutants that are able to suppress filamentation under both conditions of low magnesium as well as plasmid overexpression of QueE (see Methods for further details). From this screen we identified three different mutations—two in *ftsA* (corresponding to substitutions N170S and R126C in FtsA) and one in *ftsZ* (corresponding to D234G in FtsZ; Supplementary Fig. 11, Supplementary Table 1). We also tested the effects of expressing FtsA* (FtsA R286W), which has been shown to bypass the requirement for the division proteins ZipA^37^, FtsK^38^ and FtsN^39^, as well as the effects of increased expression of FtsP (SufI), which also suppresses the septation defects caused by mutations in certain essential division genes^40^. Expression of either FtsA* or FtsP from plasmids suppressed filamentation of *E. coli ΔmgrB* (Supplementary Fig. 12). Taken together, these data suggest that QueE inhibits a step in divisome maturation.

### YFP-QueE co-localizes with FtsZ-rings

**Figure 5:**
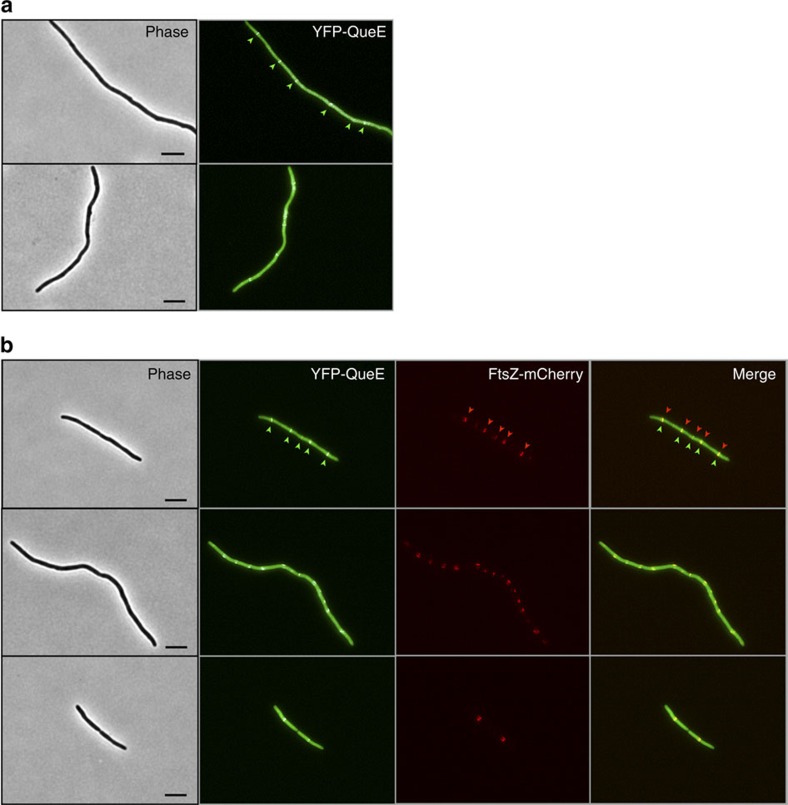
QueE localizes at the Z-ring in *E. coli* filaments. (**a**) Two representative phase contrast and fluorescence micrographs showing cellular localization of YFP-QueE expressed from a plasmid in *ΔqueE* cells (SAM144/pSY76). Cultures were grown in minimal medium containing 1 mM Mg^2+^ and ampicillin (50 μg ml^−1^) to an OD_600_=0.2–0.3 and observed by microscopy (see Methods for details). Arrows in one of the micrographs point to spots of increased YFP fluorescence. (**b**) Three representative phase contrast and fluorescence micrographs showing co-localization of FtsZ-mCherry and YFP-QueE expressed from plasmids pEG4 and pSY76, respectively, in *ΔqueE* cells (SAM144). The arrows in one set of images point to the spots of increased YFP and mCherry fluorescence. Cultures were grown in minimal medium with 1 mM Mg^2+^, ampicillin (50 μg ml^−1^) and chloramphenicol (25 μg ml^−1^) to an OD_600_=0.2–0.3 and observed by microscopy. Scale bar, 5 μm.

To test if QueE might act directly at the divisome, we constructed a translational fusion, YFP-QueE, expressed from an IPTG-inducible promoter on a plasmid, and examined the localization of this protein in a *ΔqueE* strain. Cells expressing YFP-QueE formed filaments, indicating that this function of QueE was preserved in the fusion protein (queuosine biosynthesis was not tested). Fluorescence microscopy of cell filaments showed a striped banding pattern of YFP, reminiscent of FtsZ-rings, on top of a background of diffuse YFP fluorescence throughout the cell (Fig. 5a). To simultaneously observe the localization patterns of both QueE and FtsZ, we used an mCherry-tagged FtsZ^41^. Cells expressing both fusion proteins showed that YFP-QueE co-localized with mCherry-labelled Z-rings (Fig. 5b). YFP and mCherry fluorescence images of cells expressing either YFP-QueE or FtsZ-mCherry alone showed that there was no bleed-through of the YFP signal in the mCherry channel and vice versa (Supplementary Fig. 13). We then tested the YFP-QueE localization pattern in the spontaneous suppressors that we had isolated, *ftsA-N170S*, *ftsZ-D234G* and *ftsA-R126C*. Cells with either the *ftsA-N170S* or *ftsZ-D234G* allele and containing the YFP-QueE expression plasmid were similar in size to wild-type cells (without a plasmid), and did not show any detectable spots of increased YFP fluorescence (Supplementary Fig. 14a,b). For the suppressor *ftsA-R126C*, the majority of the cells were also similar in size to wild-type cells and again had uniform YFP. However, we did observe a few filamentous cells that showed foci of increased YFP fluorescence (Supplementary Fig. 14c). We also verified that YFP-QueE expression and stability in the suppressor strains and the wild-type strain are comparable (Supplementary Fig. 15). Taken together, our results suggest that QueE localization to the Z-ring is important for filamentation and that inhibition of septation likely occurs through a direct interaction with a component of the divisome.

## Discussion

**Figure 6:**
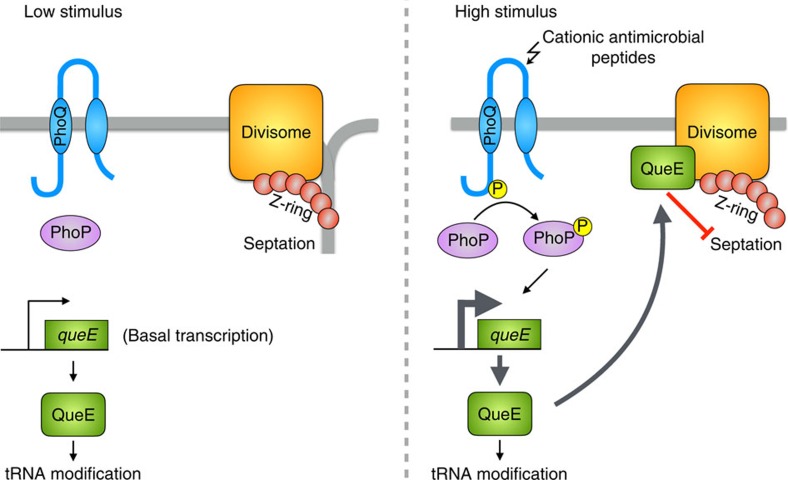
*E. coli* PhoQ/PhoP two-component system modulates cell division via QueE. PhoQ repression by high extracellular magnesium leads to low levels of phosphorylated PhoP (PhoP-P) and basal expression of QueE, sufficient for QueE's function in the queosine tRNA modification pathway. Septation is unaffected. Strong stimulation of PhoQ by cationic antimicrobial peptides leads to an increase in levels of PhoP-P, which results in increased transcription of *queE*. When cells produce high levels of QueE, the protein associates with the divisome and inhibits septation.

The filamentation described here is not a result of antimicrobial peptide-induced envelope damage or other toxic effects of the peptides. Rather, the septation block is caused by high-level activation of the PhoQ/PhoP signalling system that is induced by the antimicrobial peptides^6^ (Fig. 6). We find that growth in medium with low levels of divalent cations, another condition that stimulates PhoQ^4^, produces similarly high levels of PhoQ activation in strains that lack the negative regulator MgrB, and also leads to filamentation. By isolating and analysing suppressor mutations, we determined that the cell division block is caused by an increase in transcription of *queE*, a gene encoding an enzyme in the queuosine tRNA modification pathway. QueE along with other enzymes required for the initial steps of queuosine biosynthesis (QueC, QueD, QueF) were first identified in *Bacillus subtilis* by comparative genomics combined with experimental validation in *Acinetobacter baylyi*^31^. *B. subtilis* QueE was subsequently studied in detail and shown to be a CDG synthase^22^,^23^,^32^,^33^ (Supplementary Fig. 8). Homologues of QueE, QueD and QueC are present in *E. coli* and their role in queuosine biosynthesis has been confirmed^42^,^43^,^44^. However, we find that QueE-induced filamentation does not depend on QueD and QueC. Thus, it appears that *E. coli* QueE has another role in addition to queuosine biosynthesis. It is also noteworthy that *E. coli* QueE shares only 20% identity at the amino-acid sequence level with the *B. subtilis* QueE (and shares only 40% similarity—based on a T-Coffee alignment^45^). In addition, the genes *queC*, *queD*, *queE* are not co-transcribed in *E. coli*, and are in fact somewhat dispersed around the *E. coli* genome, in contrast with their organization in a single operon in *B. subtilis*. These observations suggest a significantly different evolutionary history for *E. coli* QueE compared with its better-studied homologue from *B. subtilis*, consistent with an additional function for the *E. coli* protein.

The localization of YFP-QueE to the divisome in filamenting cells, and the absence of this localization in cells for which filamentation has been suppressed by mutations in *ftsA* or *ftsZ*, suggests that filament formation depends on a direct interaction between QueE and divisome components. The YFP-QueE localization in cell filaments is always observed on top of considerable diffuse cytoplasmic YFP-QueE fluorescence. This observation is consistent with the fact that filamentation emerges only when *queE* expression is increased above the significant basal expression level that is independent of PhoQ/PhoP (Fig. 4d), and suggests QueE binds the divisome with low affinity. Hence, only under conditions of strong PhoQ/PhoP stimulation are QueE levels high enough to bind the divisome and inhibit septation.

In filamenting conditions, we find that the distributions of cell/filament lengths are highly heterogeneous, with the average length increasing with QueE expression. Furthermore, for less-severe filamentation, many small cells are also produced (Supplementary Table 1). We note that septation must occur at a low but non-zero frequency in filamenting cells since otherwise the cultures would consist of only a small number of extremely long cells. The wide distribution of cell lengths and the appearance of small cells suggest a model in which the locations of infrequent septation events are randomly distributed along the length of the cell filaments. Small cells will be produced when septation occurs near the filament ends; the proportion of such events will be higher when the average filament length is shorter. Taken together, these observations suggest that PhoQ/PhoP tunes the frequency of septation by adjusting *queE* expression in response to the level of stimulus.

A variety of stresses cause *E. coli* and related bacteria to grow as filaments. Examples include DNA damage, exposure to antimicrobial compounds, high hydrostatic pressure, and osmotic stress (reviewed in^46^). In some cases, the block in septation is caused by a stressor directly inhibiting a component of the cell division machinery. For example, some beta-lactam antibiotics inhibit penicillin-binding protein 3 (FtsI)^47^. In other cases, the septation block is caused by a regulatory circuit that has detected the stressor or associated environmental condition^48^. This control of division by the cell is generally assumed to increase fitness in the presence of the stress. However, the selective advantage is not always easy to discern. One well-studied example is the *E. coli* SOS response, in which detection of DNA damage leads to upregulation of the cell-division inhibitor SulA^26^,^27^. SulA-dependent filamentation is often described as a division checkpoint that protects cells from the potentially lethal consequences of initiating septation before damaged chromosomes have been repaired and segregated. However, such a role for SulA has been questioned^49^ and, to our knowledge, a protective effect of filamentation in the context of the SOS response has yet to be established. Nevertheless, there is some evidence that SulA-dependent filamentation improves survival in the face of various stresses^27^,^48^,^50^,^51^. The filamentation controlled by QueE via the PhoQ/PhoP system may similarly provide a protective function. The effects of cationic antimicrobial peptides in some environments could be more damaging for septating cells, in which case blocking septation would increase fitness. It is also possible that a selective advantage conferred by QueE-dependent filamentation is not directly associated with the PhoQ stimulus, but instead provides protection against other stresses that are typically encountered in environments where *E. coli* naturally experiences high levels of cationic antimicrobial peptides or other stimuli that strongly activate PhoQ. With either scenario, this newly-discovered pathway controlling division may provide an important mechanism for cells to tune the frequency of septation to maximize their chance of survival.

## Methods

### Strains and plasmids

See Supplementary Table 2 for a list of strains and plasmids and Supplementary Table 3 for a list of primers used in the study. All strains were derived from *E. coli* K-12 MG1655 except EC448 and JNC19, which were derived from MC4100. Strain EC448 and plasmid pDSW914 were gifts from Dr David Weiss; pBAD33-ftsA* was a gift from Dr William Margolin; strain GS0241 was a gift from Dr Gisela Storz; plasmid pEG4 was a gift from Dr Kenn Gerdes; and plasmids pGB2 and pLPQ*2 were gifts from Dr Carey Waldburger. Gene deletions and reporter constructs were transferred between strains using transduction with P1_vir_. Deletions in the Keio collection^30^ that were used for strain construction were confirmed by PCR using primers flanking the targeted gene. Strain GS0241 was used to construct a *yqcG* deletion by P1_vir_ transduction. Kanamycin resistance markers were excised, when required, using the FLP-recombinase-expressing plasmid pCP20^52^,^53^. Plasmid pRL03 carrying *queE* under the control of the *trc* promoter was constructed by cloning a PCR-amplified *queE* insert from the *E. coli* MG1655 genome into pEB52 via EcoRI and BamHI restriction sites. The *queE-yfp* reporter construct, which is an operon fusion of *yfp* to the *queE* gene, was made by integrating *yfp* downstream of *queE*. To construct this reporter, the *yfp-kan* cassette of pEB112 was amplified by PCR using primers queE-yfp-lred-U1 and queE-yfp-lred-L1 and integrated into the chromosome downstream of *queE* by recombineering^53^. The resulting transcriptional fusion has an intergenic region of 50 bp between *queE* and *yfp*, and includes a ribosome binding site for *yfp*. The operon fusion was then transduced into a clean MG1655 and various mutant strains. Plasmid pSY76 encoding a YFP-QueE translational fusion under the control of the *trc* promoter was constructed by three-way ligation. DNA fragments sacI-yfp-speI and xbaI-queE-bamHI were amplified from pEB112 and pRL03, respectively, and ligated with sacI-bamHI cut-pEB52. All constructs were confirmed by DNA sequencing.

### Media and growth conditions

Liquid cultures were grown at 37 °C with aeration, unless otherwise indicated, in either LB Miller medium (Fisher Scientific) or in minimal A medium^54^ containing 0.2% glucose, 0.1% casamino acids and the indicated concentration of MgSO_4_. For routine growth on solid medium, LB or minimal medium containing bacteriological grade agar (Fisher Scientific) was used. The antibiotics ampicillin, kanamycin, chloramphenicol and spectinomycin were used at concentrations 50–100, 25, 20–25 and 50 μg ml^−1^, respectively. The antibacterial peptide C18G (Anaspec) was added to a final concentration of 9 or 7 μg ml^−1^, as indicated. The *lac* and *trc* promoters were induced with the indicated concentrations of β-isopropyl-D-thiogalactoside (IPTG) and the *araBAD* promoter was induced with 30 mM arabinose.

### Microscopy

Prior to each experiment, strains were grown in liquid cultures in minimal medium containing 1 mM Mg^2+^ to saturation, overnight at 37 °C with aeration. Saturated cultures were then diluted 1:1,000 into fresh medium with 1 mM, 10 mM or no added Mg^2+^, and inducers were added as indicated. Cells were grown to an OD_600_ of ∼0.2–0.3 (about 4 h). For measurements of CFP, YFP fluorescence from transcriptional reporter strains, cultures were rapidly cooled in an ice-water slurry and streptomycin was added to a final concentration of 250 μg ml^−1^ to stop protein synthesis. Microscope slides were prepared with 1% agarose pads and cellular fluorescence was measured by microscopy and quantified with software written in LabView (National Instruments)^19^,^55^. Background fluorescence, measured from the strain MG1655 cultured under the same conditions as the reporter strains, was subtracted from each fluorescence measurement. Average cell lengths were determined from phase contrast images using ImageJ^56^ and the MicrobeJ plugin^57^. For antimicrobial peptide experiments, saturated cultures grown in minimal medium were diluted 1:1,000 into fresh medium containing 0.1 mM Mg^2+^ with (or without) C18G peptide to a final concentration of 9 or 7 μg ml^−1^ as indicated. Cells were grown to an OD_600_ ∼0.2–0.3 (about 5–6 h for cells growing with C18G), and analysed by microscopy. For strains containing either pGB2 or pLPQ*2, saturated cultures were prepared by overnight growth in minimal medium containing 50 μg ml^−1^ spectinomycin, followed by 1:1,000 dilution in fresh medium for growth and microscopy as described above. For membrane labelling, a 1 mg ml^−1^ stock solution of FM4–64 in DMSO was diluted 1:10,000 into cultures grown to early exponential phase 1 h prior to microscopy. For DNA staining, a 1 μg ml^−1^ stock solution of DAPI in 100 mM phosphate buffer, pH 7, was prepared. A 100-fold dilution of this DAPI stock was used to stain cells for 10 min at 25 °C. Cells were applied to microscope slides containing an agarose pad and visualized by fluorescence microscopy. For FRAP experiments, strains expressing YFP from a P_*mgrB*_*-yfp* reporter construct were grown to early exponential phase in minimal medium with no added Mg^2+^ and immobilized on microscope slides containing agarose pads. FRAP and image acquisition was carried out using a DM4000 spinning disk confocal microscope (Leica) with a × 100 1.4 numerical aperture objective, an XY Piezo-Z stage (Applied Scientific Instrumentation), and a laser merge module equipped with a 488 nm Argon ion laser (Spectral Applied Research) controlled by MetaMorph software (Molecular Devices). Images were acquired with an EMCCD camera (Hamamatsu Photonics) before bleaching, immediately after bleaching, and then every 2 s for 30 s. The extent of photobleaching that occurred during imaging was ∼25–30% over the entire time course, as estimated by analysis of unbleached control cells.

For FtsZ-GFP localization experiments, saturated overnight cultures of EC448 and JNC19 were diluted 1:1,000 into fresh medium containing 20–50 μM IPTG and grown at 37 °C with aeration for 6 h. FtsZ-rings were visualized by fluorescence microscopy. Measurements of the mean distance between Z-rings in filamenting cells were made using the MicrobeTracker suite for MATLAB^58^. For imaging the localization of YFP-QueE and FtsZ-mCherry, the strain SAM144 was transformed with either pSY76 alone, pEB52 and pEG4, or pSY76 and pEG4 (ref. 41). Saturated cultures of SAM144/pSY76, SAM144/pSY76/pEG4 and SAM144/pEG4/pEB52 were diluted 1:500 in fresh minimal medium with 50 μg ml^−1^ ampicillin and/or 20 μg ml^−1^ chloramphenicol, and grown until an OD_600_ ∼0.2–0.3. For YFP-QueE expression, basal levels of protein from plasmid pSY76 were sufficient while FtsZ-mCherry expression was induced using 0.5% arabinose for 1 h. For localization of YFP-QueE in spontaneous suppressors of filamentation, strains SAM169, SAM170 and SAM171 were transformed with pSY76. The resulting strains were inoculated in minimal medium containing 50 μg ml^−1^ ampicillin, grown overnight with aeration at 37 °C and diluted 1:500 in fresh minimal medium with 50 μg ml^−1^ ampicillin, and grown until an OD_600_ ∼0.2–0.3. Cellular localization of YFP-QueE and FtsZ-mCherry were observed by live-cell microscopy on agarose pads as outlined above.

### Plasmid complementation

For plasmid expression of MgrB and QueE, cells containing either pAL8, pRL03, pEB52 or pTrc99a grown overnight in minimal medium containing 1 mM Mg^2+^ and 50 μg ml^−1^ ampicillin were diluted 1:500 into fresh medium with 1 mM Mg^2+^ and 50 μg ml^−1^ ampicillin. Similarly, for complementation experiments with wild-type PhoQ, PhoQ T281R and SafA, strains containing either pTM69, pTM153, pEB52, pSafA or pTrc99a grown overnight in minimal medium containing 1 mM Mg^2+^ and 50 μg ml^−1^ ampicillin were diluted 1:500 into fresh medium. Cultures were then grown at 37 °C with aeration for 2 h, followed by induction using 0.5 mM IPTG for 3 h. For FtsA* expression, TIM92 and AML20 strains containing either pBAD-FtsA* or pBAD33 were grown overnight in minimal medium containing 1 mM Mg^2+^ and 25 μg ml^−1^ chloramphenicol. Overnight cultures were diluted 1:500 into fresh media with no added Mg^2+^ and 12.5 μg ml^−1^ chloramphenicol and grown at 37 °C with aeration for 2 h, and then induced by addition of arabinose to a final concentration of 0.5% for 3 h. For FtsP expression, strains containing either pDSW914 or pTrc99a grown overnight in minimal medium containing 1 mM Mg^2+^ and 50 μg ml^−1^ ampicillin were diluted 1:500 into fresh medium with no added Mg^2+^ and 50 μg ml^−1^ ampicillin. Cultures were grown at 37 °C with aeration for 2 h, and then induced by addition of 1 mM IPTG for 3 h. In each case, cultures were rapidly cooled in an ice-water slurry and analysed by phase contrast microscopy.

### Transposon mutagenesis screen

Transposon mutagenesis was performed with the plasmid pRL27, which carries a hyperactive Tn5 transposase gene and a mini-Tn5 transposable element encoding a kanamycin resistance gene and the conditional origin of replication *ori*R6K (ref. 59). The strain AML20 was transformed with pRL27 by electroporation and selected on low-Mg^2+^ agarose plates composed of minimal A medium with 0.2% glucose, 0.1% casamino acids, 4% SeaPlaque agarose (Lonza) and 25 μg ml^−1^ kanamycin. Following overnight incubation at 37 °C, colonies were pooled and suspended in minimal medium with no added Mg^2+^. The cells were then passed through a sterile fluted filter paper (Eaton-Dikeman Grade 515, size 12.5 cm) in order to enrich for small (non-filamentous) cells. The flow-through solution was diluted into minimal medium with no added Mg^2+^ and grown for roughly 5 h (OD_600_ of ∼0.2), re-filtered, and plated on low-Mg^2+^ agarose plates. Colonies were then screened under a magnifying glass for wild type-like morphology (Supplementary Fig. 6) and re-streaked on low-Mg^2+^ agarose plates to ensure they maintained the colony morphology and then checked by microscopy for suppression of filamentation. (Colonies were resuspended in minimal medium and examined by phase contrast microscopy.) The suppressor phenotype was confirmed by transducing the transposon insertion into a fresh AML20 background. Transposon insertion sites were identified essentially as described in ref. 60 except that sheared DNA was end-repaired using the End-It DNA repair kit (Epicentre) prior to ligation. Genomic DNA flanking the transposon was determined by sequencing the recovered plasmids using the primers *ori*R6Kseqprim1 and TnmodRkan4.

### Spontaneous suppressor screen

An overnight culture of the strain SAM4 grown in minimal medium supplemented with 0.2% glucose and 0.1% casamino acids, was diluted 1:1,000 in fresh medium containing no added Mg^2+^. Cells were grown for 5 h with aeration at 37 °C and plated on low-Mg^2+^ agarose (4% Seaplaque) plates. Large colonies (indicating suppression of the growth defect of SAM4 on low Mg^2+^) that also had a morphology similar to colonies of non-filamenting cells were selected. After re-streaking and verifying that the colony phenotype was preserved, cells were checked by microscopy for suppression of filamentation. To identify mutations that act downstream of QueE, suppressors were transformed with pRL03 and tested for their ability to suppress filamentation from overexpression of QueE. Overnight cultures of strains containing pRL03 were diluted 1:500 in fresh minimal medium, grown for 3 h followed by addition of 1 mM IPTG to induce QueE expression and grown for another 3 h before performing microscopy to identify suppressors. Three suppressors were identified. Kanamycin insertions linked to the suppressor mutations were then constructed as follows. For each mutant, a pool of random mini-Tn5 insertions was prepared as described above and used to transduce kanamycin resistance to a fresh strain background by P1 transduction. Kanamycin resistant colonies were screened for filamentation suppression as described above. Several such colonies were selected and confirmed to give P1 lysates that, when transduced to a fresh strain background, suppressed filamentation at a high frequency. The positions of the transposon insertions were then determined as described above and found to be near the *ftsQAZ* locus. The specific mutations in two of the suppressors were identified by whole-genome sequencing. Genomic DNA was extracted using a Qiagen DNeasy Blood and Tissue Kit and eluted in 10 mM Tris-Cl pH 8.5. DNA sequencing (Illumina Miseq platform) was performed by the Center For AIDS Research SGA Service Center at the University of Pennsylvania. Single nucleotide polymorphisms were determined using the Freebayes tool on Galaxy^61^,^62^,^63^ for the parent strain and each suppressor, and were confirmed by DNA sequencing. The mutation in the third suppressor was identified through PCR amplification of the *ftsQAZ* locus and sequencing using primers specific to genes *ftsQ*, *ftsA* and *ftsZ*.

### PhoP purification and gel-shift assays

PhoP-His_6_ was expressed from pTM50 in *E. coli* strain BL21(DE3) and purified by Ni-NTA affinity chromatography. Purity of the eluted fractions was determined by SDS–polyacrylamide gel electrophoresis and fractions containing the highest amount of protein were pooled and dialysed overnight at 4 °C in a buffer containing 50 mM Tris-Cl pH 7.5, 100 mM KCl, 1 mM MgCl_2_, 1 mM DTT and 20% glycerol. The protein was further concentrated in fresh dialysis buffer containing 50% glycerol for 4 h at 4 °C and 50 μl aliquots were stored at −80 °C. Final protein concentration was estimated using the BCA Protein Assay Kit (Pierce). PhoP was phosphorylated *in vitro* by acetyl phosphate for 1 h at 37 °C (ref. 64). Excess acetyl phosphate was removed from phosphorylated PhoP-His_6_ by spinning through a G-25 MicroSpin column (GE Healthcare) that was pre-equilibrated with 50 mM Tris-Cl pH 7.5. Phosphorylated PhoP-His_6_ was stored on ice and used within 2 h. DNA sequences for EMSAs were amplified by PCR from *E. coli* MG1655 genomic DNA using primers for P_*mgrB*_ (1906801-1907100), X1 (2903441-2903723), X2 (2903720-2904019) and P_*lacZ*_ (365450-365743). (Each forward primer contains an *Eco*RI site at the 5′-end.) PCR products were purified by agarose gel electrophoresis. To synthesize radioactive probes, DNA fragments were digested with EcoRI, purified (Qiagen PCR purification kit) and 100 ng of DNA was used to perform a fill-in reaction with Klenow polymerase, dGTP, dCTP, dTTP and α-^32^P dATP for 20 min at 25 °C. Following this step, samples were passed through Sephadex G-25 spin columns (GE Healthcare) to remove excess label and for buffer exchange. Binding reactions were performed in 20 μl with 10 fmol of labelled DNA probe, phosphorylated PhoP-His_6_, and 50 ng μl^−1^ poly [dI-dC] in a buffer containing 50 mM Tris-Cl pH 7.5, 50 mM KCl, 1 mM DTT, 50 μg ml^−1^ BSA and 5% glycerol, for 20 min at 25 °C. Five microlitres of each sample was mixed with 1 μl of 6 × DNA loading dye (40% sucrose, 0.25% bromophenol blue, 0.25% xylene cyanol) and run on a non-denaturing polyacrylamide gel (5%) in 0.5X Tris-acetate-EDTA running buffer at 150 V for 3 h. Following electrophoresis, the gel was dried under vacuum for 1 h at 80 °C, exposed overnight to a phosphorimager screen (Molecular Dynamics) and imaged with a Typhoon scanner (GE Healthcare).

### Western blot analysis

Cells from saturated cultures were harvested and resuspended in 30 mM Tris-Cl pH 8.0, 50 mM DTT and 1X NuPAGE (Invitrogen) LDS loading buffer. The samples were boiled for 10 min and loaded on 7% Tris-acetate gel (NuPAGE, Invitrogen). Proteins were transferred to Immobilon-P PVDF (Millipore) followed by western blot analysis. YFP was detected with rabbit polyclonal anti-GFP antibodies (Living Colors A.v. Peptide Antibody #632377, Takara Clontech) diluted 1:400 to a final concentration of 0.25 μg ml^−1^. A horseradish peroxidase-conjugated donkey anti-rabbit antiserum (GE Healthcare #NA934) diluted 1:25,000 was used as the secondary antibody.

### Data availability

The authors declare that all data supporting the findings of this study are available within the article and its Supplementary Information files.

## Additional information

**How to cite this article:** Yadavalli, S. S. *et al*. Antimicrobial peptides trigger a division block in *Escherichia coli* through stimulation of a signalling system. *Nat. Commun.* 7:12340 doi: 10.1038/ncomms12340 (2016).

## Supplementary Material

Supplementary Information Supplementary Figures 1-15, Supplementary Tables 1-3 and Supplementary References
