# Supplementary material for: Antimicrobial peptides trigger a division block in Escherichia coli through stimulation of a signalling system
Source: Nat Commun. 2016 Jul 29;7:12340. doi: 10.1038/ncomms12340 (PMC4974570; doi:10.1038/ncomms12340)
Supplement: Supplementary Information — Supplementary Figures 1-15, Supplementary Tables 1-3 and Supplementary References [file ncomms12340-s1.pdf]

Supplementary Information

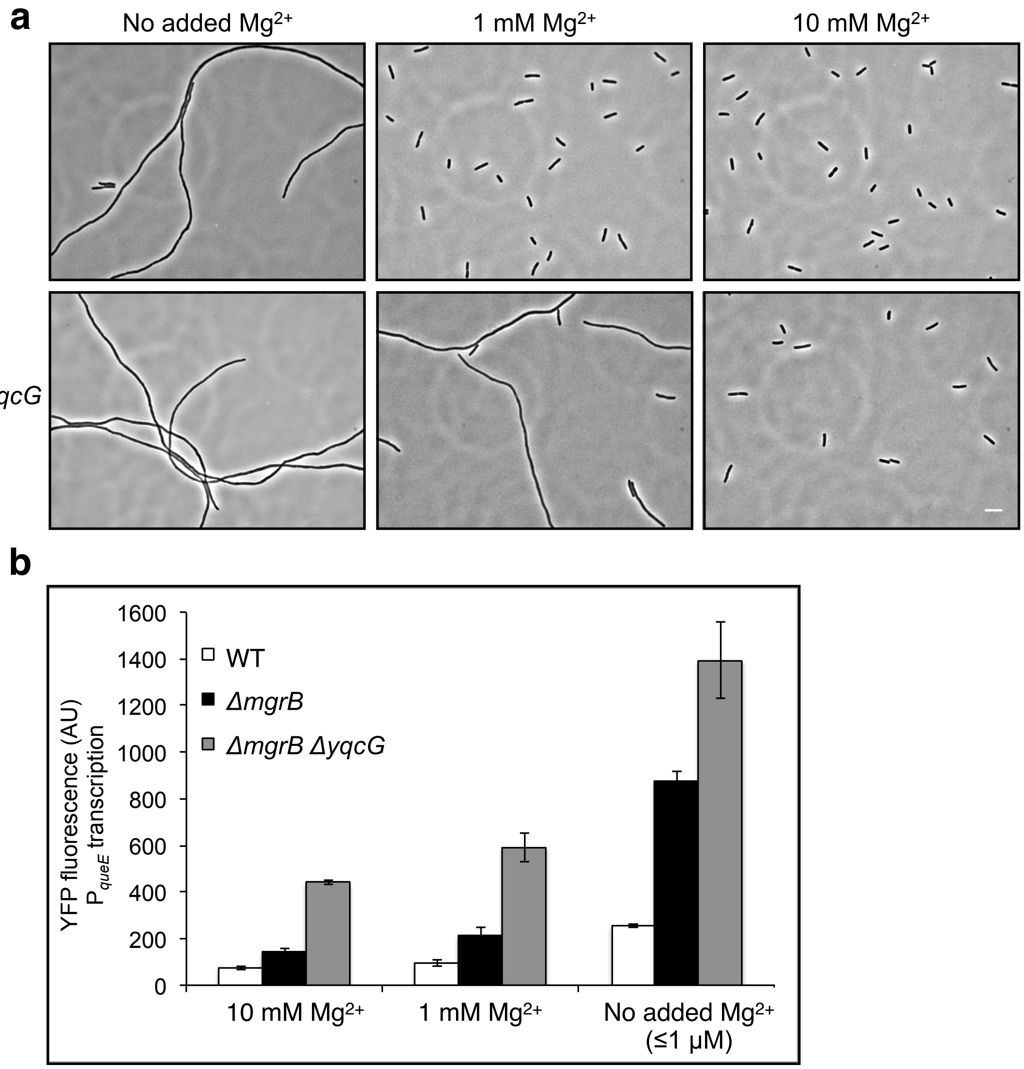

Supplementary Figure 1. Filamentation and *queE* transcription in *ΔmgrB* and *ΔmgrB ΔyqcG* cells. a) Phase contrast images of *Δ mgrB* (AML20) and *ΔmgrB ΔyqcG* (SAM4) cells grown in the indicated concentrations of Mg^{2+}. **b)** YFP fluorescence measured from a *queE-yfp* operon fusion in wild-type (SAM54), *Δ mgrB* (SAM55) and *ΔmgrB ΔyqcG* (SAM56) strains in minimal medium at the indicated Mg^{2+}

concentration. Data represent means and standard deviations from at least three independent experiments. Scale bar = 5 μm.

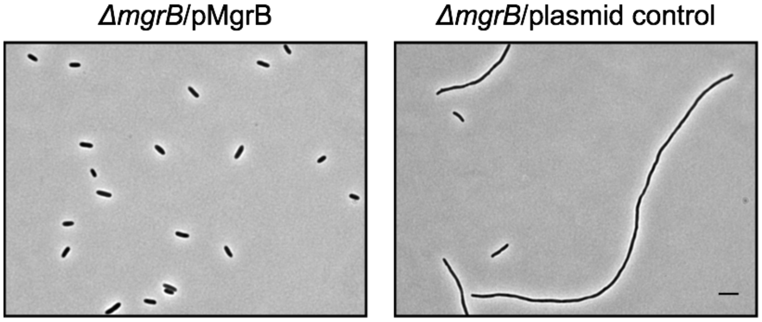

Supplementary Figure 2. A plasmid-borne copy of *mgrB* complements *ΔmgrB* . Phase contrast images of a *ΔmgrB* strain containing either an *mgrB* expression plasmid pMgrB (pAL8) or a control plasmid (pEB52) grown in minimal medium with no added Mg^{2+} and with ampicillin (50 μg ml^{-1}), induced with 0.5 mM IPTG for 3 h. Scale bar = 5 μm.

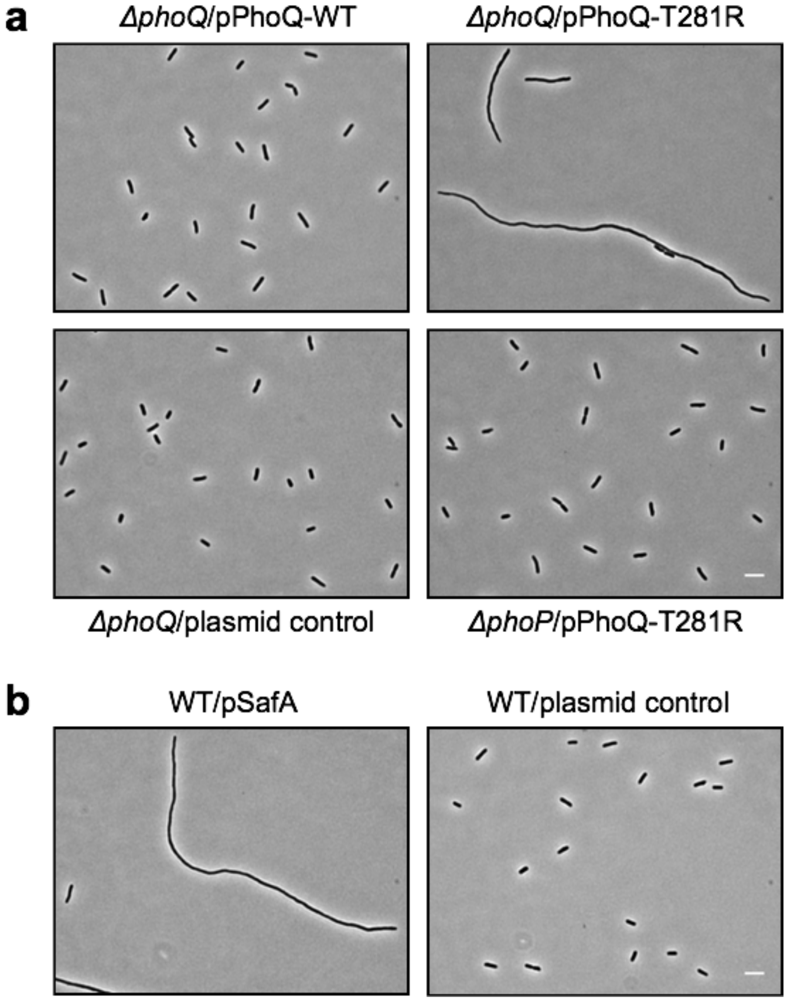

Supplementary Figure 3. Filamentation from high PhoQ stimulation is not dependent on low Mg^{2+} or antimicrobial peptides. a) Effect on cellular morphology from expressing a kinase+ phosphatase- PhoQ variant, PhoQ T281R. Increased expression of PhoQ T281R produces high-level activation of the PhoQ/PhoP system, in contrast with increased expression of wild-type PhoQ[1]. The strains and plasmids are as follows: *ΔphoQ* (TIM100), *ΔphoP* (TIM233), pPhoQ-WT (pTM69), pPhoQ-T281R (pTM153), plasmid control (pEB52). **b)** Cellular morphology from inducing the PhoQ-stimulating connector protein SafA. The strain is TIM210 and the control plasmid is

pTrc99a. Cultures were grown in minimal medium with 1 mM Mg^{2+} and 50 μg ml^{-1} ampicillin. Following 2 h of growth after dilution from an overnight culture, IPTG was added to 0.5 mM (a) or 100 µM (b) and cultures were grown for an additional 3 h prior to microscopy. Scale bar = 5 µm.

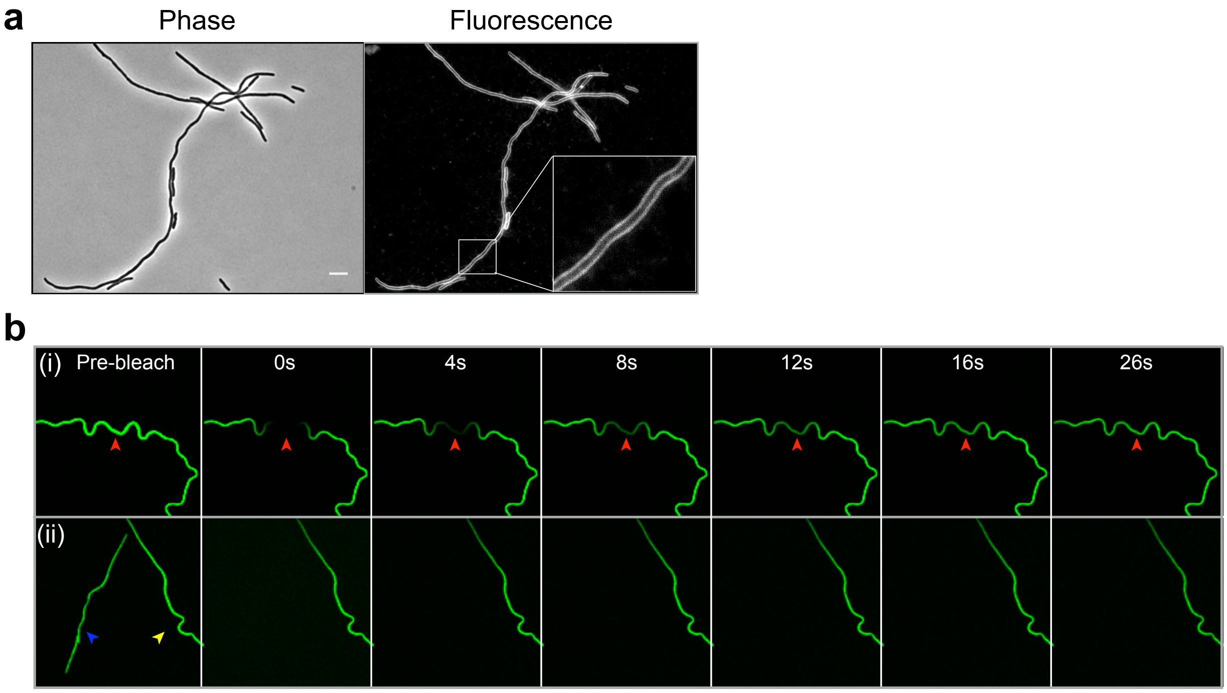

Supplementary Figure 4. Filamentous cells have a continuous cytoplasm and no visible septa. a) Phase contrast and fluorescence micrographs of *ΔmgrB* cells (AML20) labeled with the membrane dye FM4-64. The inset in the fluorescence image shows a close-up of FM4-64-stained membrane. Cultures were grown in minimal medium with no added Mg^{2+}. Scale bar = 5 μm. **b)** Fluorescence recovery after photobleaching in (i) a *ΔmgrB* cell (AML20) photobleached over a small section (denoted by a red arrow) and

monitored over time; (ii) a control cell photobleached completely (blue arrow) that does not recover any fluorescence with time, and a second cell that was not bleached (yellow arrow), which serves as a control for photobleaching during image acquisition.

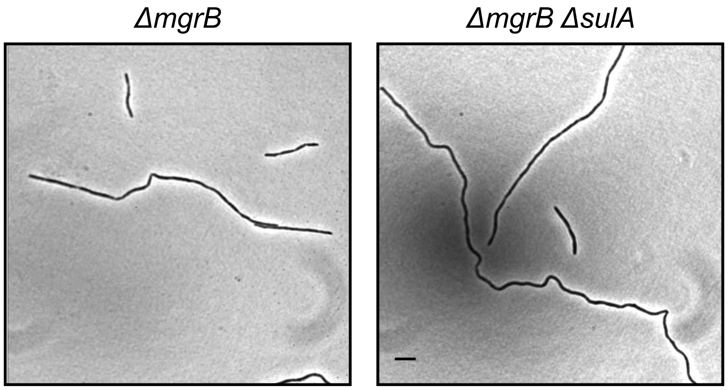

Supplementary Figure 5. Cell filamentation is SulA-independent. Phase contrast micrographs of *ΔmgrB* (AML20) and *ΔmgrB ΔsulA* (AMS3) cells grown in minimal medium with no added Mg^{2+}. Scale bar = 5 μm.

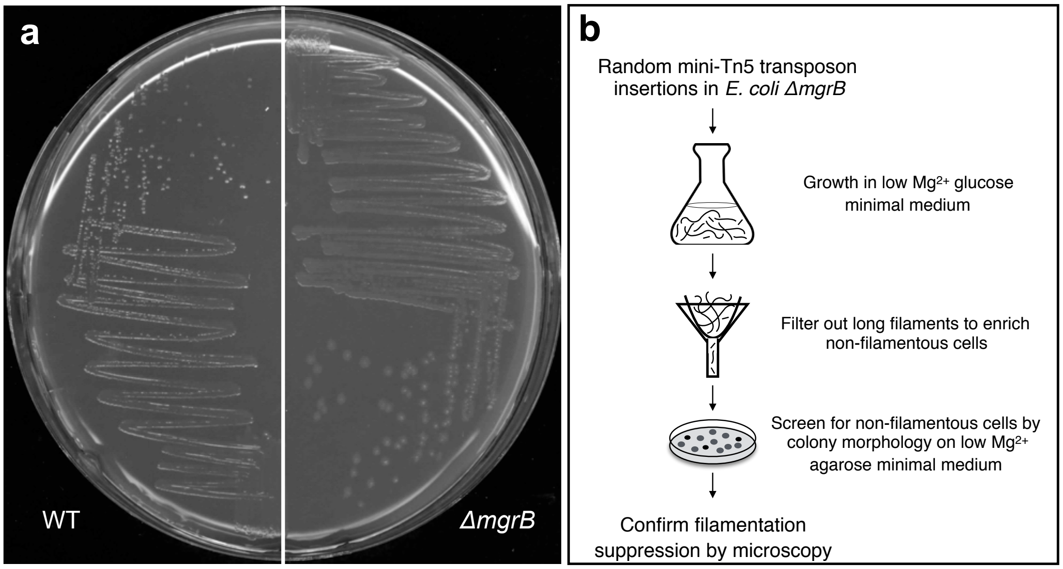

Supplementary Figure 6. Transposon insertion screen for filamentation suppressors. a) Colony morphology of wild-type, (TIM92) and *ΔmgrB* (AML20) strains on plates containing minimal medium with 4% SeaPlaque agarose and no added Mg^{2+}. **b)** Schematic representation of the screen used to identify filamentation suppressors. Briefly, a liquid culture of random transposon insertions in a *ΔmgrB* strain was passed through filter paper to enrich for non-filamentous cells, plated on low Mg^{2+} agarose plates, and screened for candidate suppressors based on colony morphology. Approximately 10,000 colonies were screened and several potential suppressors were identified. Mutants that were confirmed by microscopy to produce non-filamentous cells were then selected, and the phenotype was reassessed after the transposon insertion was moved to a clean *ΔmgrB* background by P1 transduction.

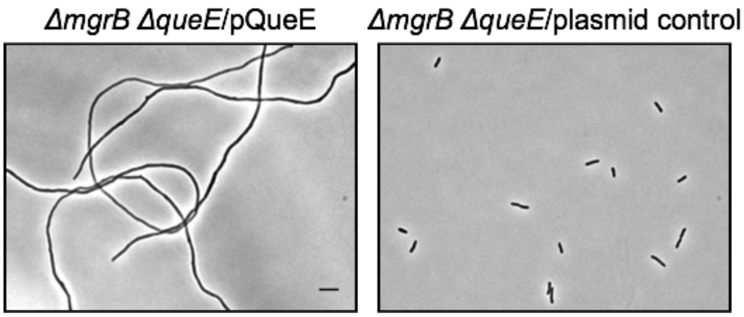

Supplementary Figure 7. A plasmid-borne copy of *queE* restores filamentation in a *ΔmgrB ΔqueE* strain. *ΔmgrB ΔqueE* cells (JNC21) harboring either a *queE* expression plasmid pQueE (pRL03) or a control plasmid (pTrc99a) grown in minimal medium with no added Mg^{2+}, with ampicillin (50 $\mu g\ ml^{-1}$), and induced with 100 µM IPTG for 3 h. Scale bar = 5 μm.

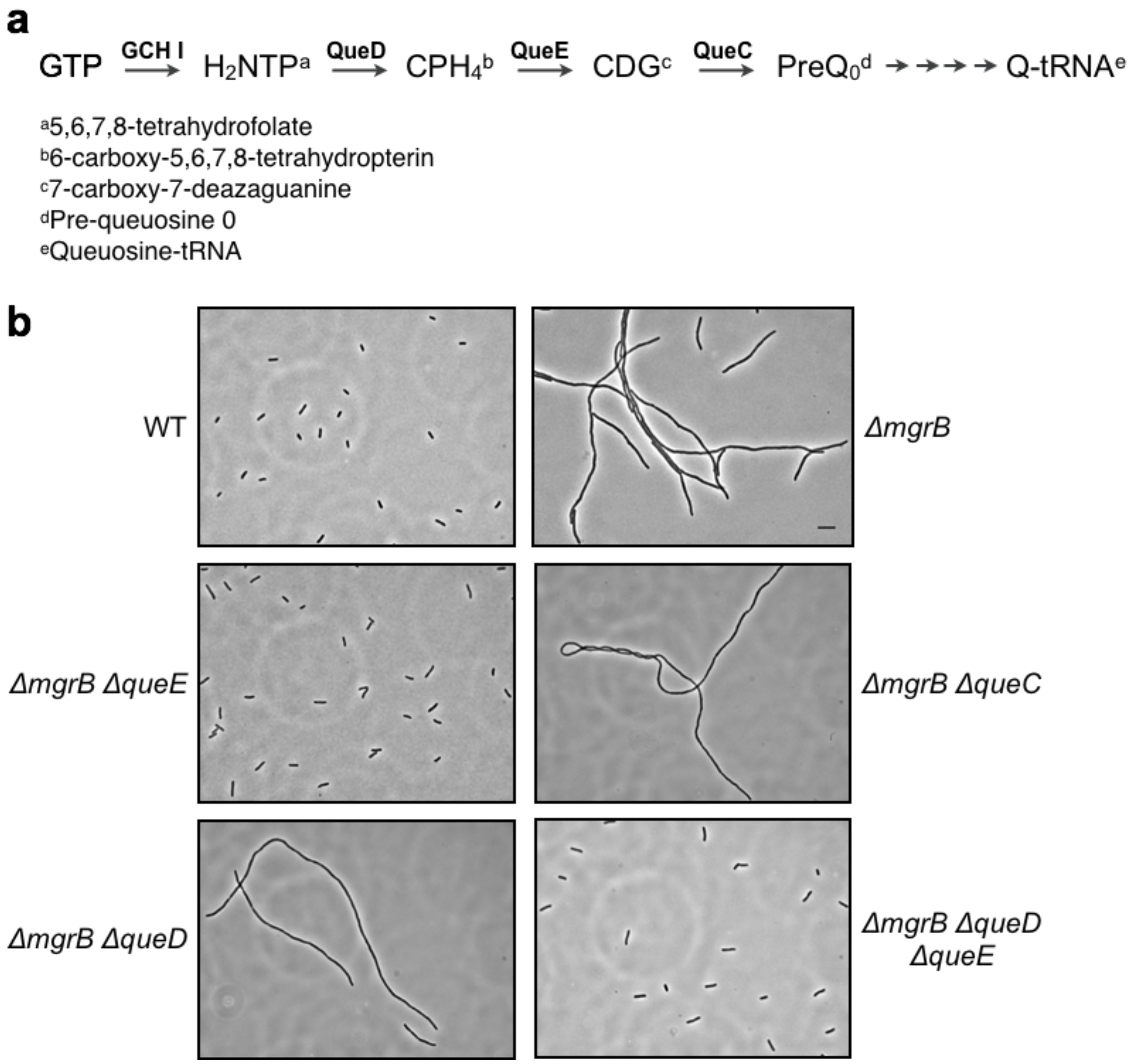

Supplementary Figure 8. Filamentation of *ΔmgrB* strains is independent of the steps in the queuosine biosynthesis pathway. a) Schematic representation of the queuosine biosynthesis pathway (see ref.[2]). **b)** Phase contrast micrographs of wild-type (TIM92), *ΔmgrB* (AML20), *ΔmgrB ΔqueE* (JNC21), *ΔmgrB ΔqueC* (RL2), *ΔmgrB ΔqueD* (RL4) and *ΔmgrB ΔqueD ΔqueE* (MMR161) cells grown in minimal medium with no added Mg^{2+}. Scale bar = 5 μm.

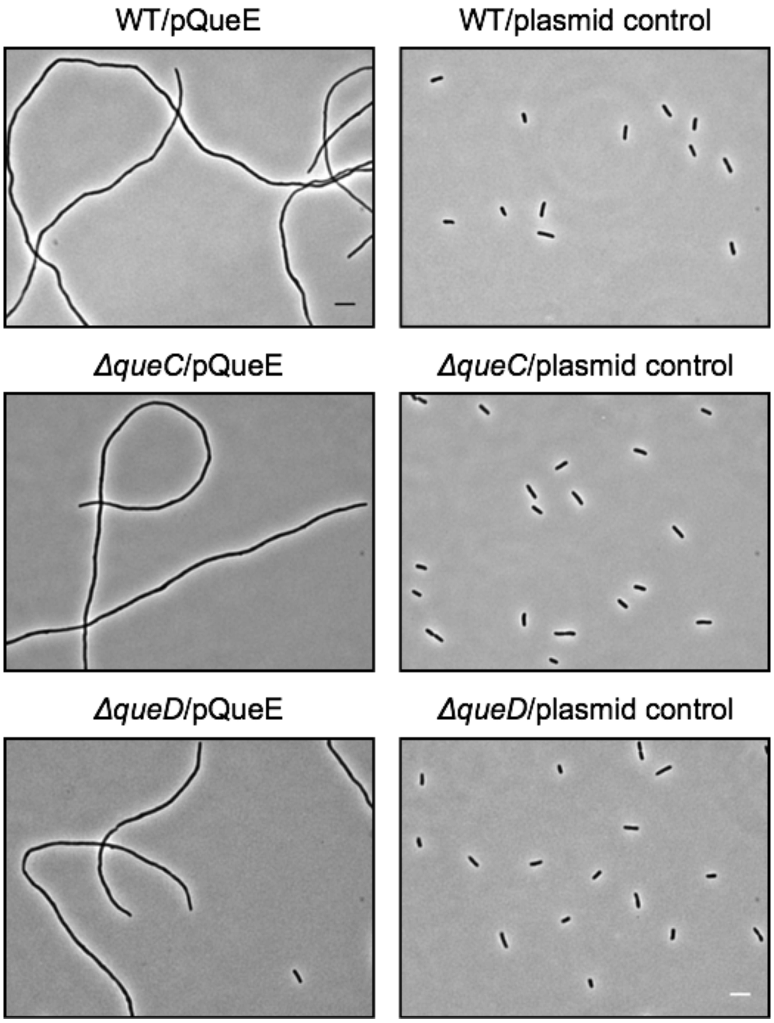

Supplementary Figure 9. Increased expression of *queE* leads to filamentation. Wild-type (TIM92), *ΔqueC* (RL3), and *ΔqueD* (RL1) cells harboring either a *queE* expression plasmid pQueE (pRL03) or a control plasmid (pEB52) were grown in minimal medium with 1 mM Mg^{2+}, with ampicillin (50 μg ml^{-1}), and induced with 0.5 mM IPTG for 3 h. Scale bar = 5 μm.

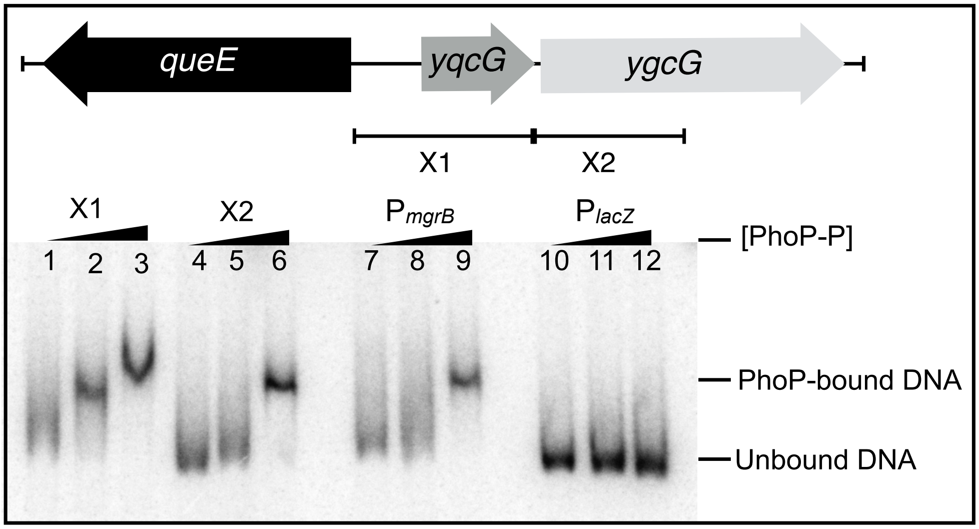

Supplementary Figure 10. EMSAs demonstrate that PhoP binds to a region upstream *queE*. X1 consists of a 300 bp region upstream of the *queE* start codon and X2 consists of a second 300 bp region upstream of X1. P_{mgrB} and P_{lacZ} denote the *mgrB* and *lacZ* promoters, respectively, which were included as positive and negative controls. Lanes 1, 4, 7, 10 contain no PhoP-P, lanes 2, 5, 8, 11 contain ~3 μM PhoP-P, and lanes 3, 6, 9,12 contain ~6 μM PhoP-P.

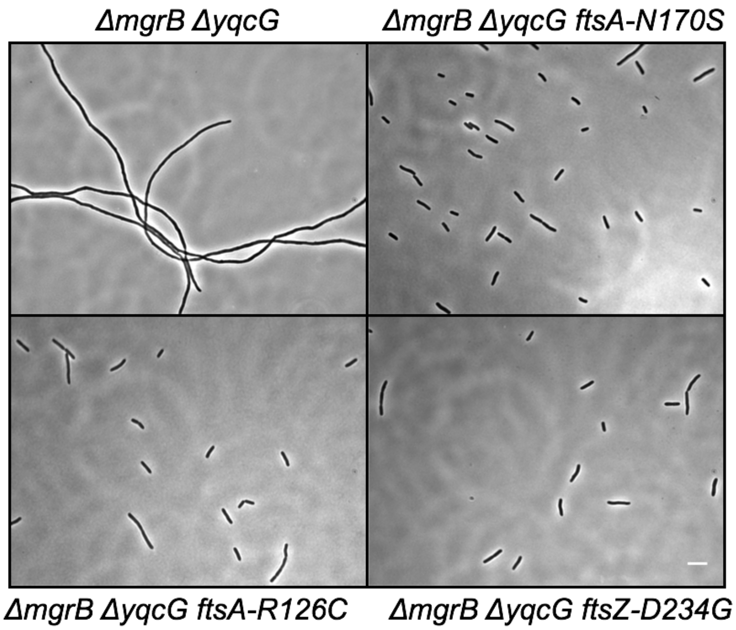

Supplementary Figure 11. Mutations in the genes of cell division components suppress filamentation. Phase contrast micrographs of *ΔmgrB ΔyqcG* (SAM4), and filamentation suppressors derived from this strain, *ftsA-N170S* (AIC155), *ftsA-R126C* (AIC156) and *ftsZ-D234G* (AIC157). Cells were grown in minimal medium with no added Mg^{2+}. Scale bar = 5 μm.

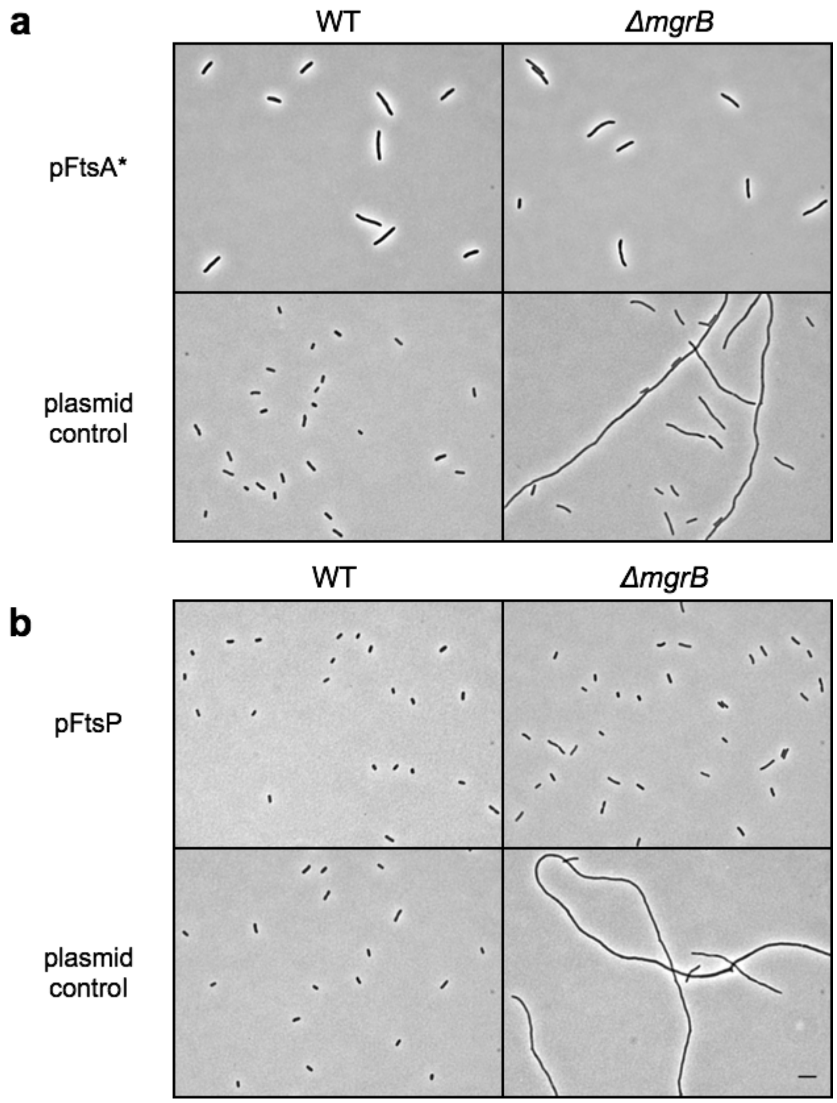

Supplementary Figure 12. Expression of FtsA* and FtsP suppress filamentation.

a) Wild-type (TIM92) and *ΔmgrB* (AML20) cells harboring either FtsA* expression plasmid pFtsA* (pBAD-FtsA*) or control plasmid (pBAD33). **b)** Wild-type (TIM92) and *ΔmgrB* (AML20) cells harboring either an FtsP expression plasmid pFtsP (pDSW914) or control plasmid (pTrc99a). Cultures were grown in minimal medium with no added Mg^{2+} containing appropriate antibiotics, and induced with either 0.5% arabinose or 0.5 mM IPTG for 3 h. Scale bar = 5 μm.

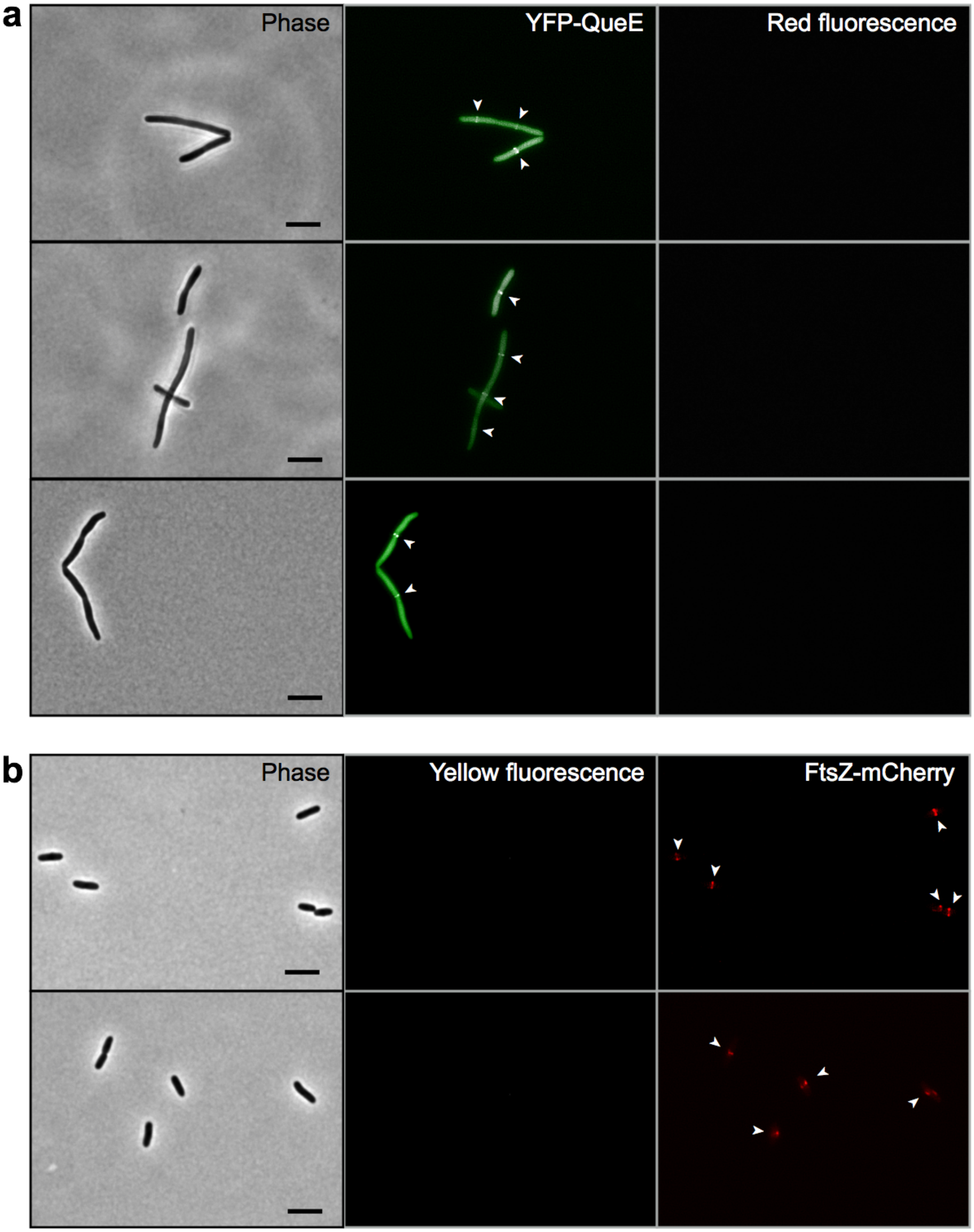

a
Phase
YFP-QueE
Red fluorescence
b
Phase
Yellow fluorescence
FtsZ-mCherry

Supplementary Figure 13. Images showing no bleed-through from the YFP to mCherry fluorescence channel and vice versa for QueE-YFP and FtsZ-mCherry. **a)** Three representative phase contrast and fluorescence micrographs showing localization of YFP-QueE expressed from plasmid pSY76 in *ΔqueE* cells (SAM144). The arrows point to the spots of increased YFP fluorescence. Red fluorescence images indicate that there was no detectable bleed-through from the yellow to red channel. Cultures were grown in minimal medium with 1 mM Mg^{2+}, and ampicillin (50 μg ml^{-1}) to an OD_{600} = 0.2-0.3. **b)** Two representative phase contrast and fluorescence micrographs showing localization of FtsZ-mCherry expressed from plasmid pEG4 in *ΔqueE* cells (SAM144). The arrows point to the spots of increased mCherry fluorescence. Yellow fluorescence images indicate that there was no detectable bleed-through from the red to yellow channel. Brightness and contrast values are similar to those in the images in Fig. 5 and Supplementary Fig. 14. Cultures were grown in minimal medium with 1 mM Mg^{2+}, ampicillin (50 μg ml^{-1}) and chloramphenicol (25 μg ml^{-1}) to an OD_{600} = 0.2-0.3, induced with 0.5% arabinose for 1 h. Scale bar = 5 μm.

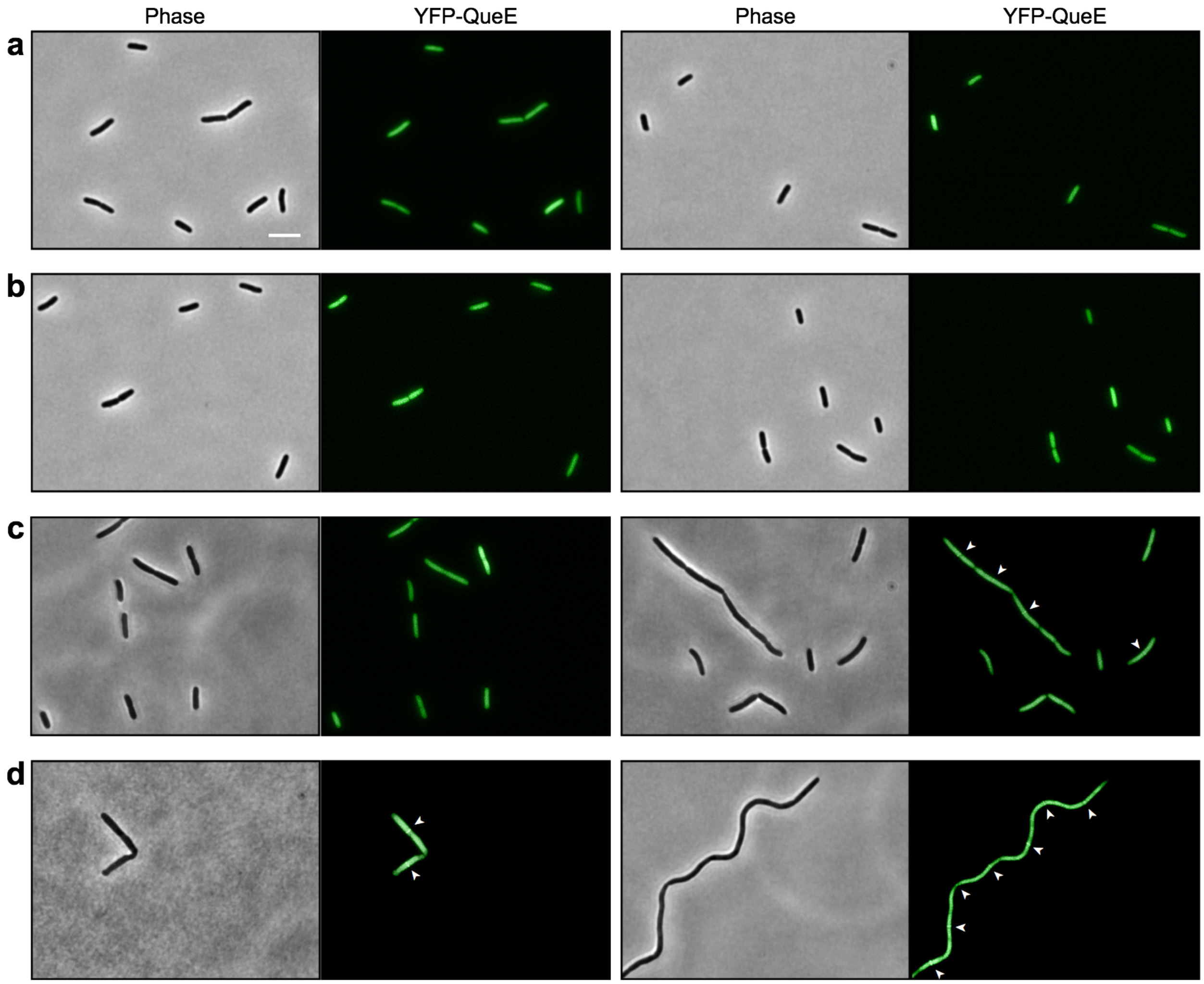

Supplementary Figure 14. QueE localization in spontaneous suppressors of filamentation. For each suppressor, two representative phase contrast and fluorescence micrographs of cells with YFP-QueE expressed from a plasmid are shown. **a)** *ΔmgrB ΔyqcG ftsA-N170S* (SAM169/ pSY76); **b)** *ΔmgrB ΔyqcG ftsZ-D234G* (SAM170/pSY76); **c)** *ΔmgrB ΔyqcG ftsA-R126C* (SAM171/ pSY76) and **d)** *ΔmgrB ΔyqcG* (SAM51/pSY76) cells. Arrows in the micrographs point to spots of increased

YFP fluorescence. All cultures were grown in minimal medium containing 1 mM Mg^{2+} and ampicillin (50 μg ml^{-1}) to an OD_{600} = 0.2-0.3. Scale bar = 5 μm.

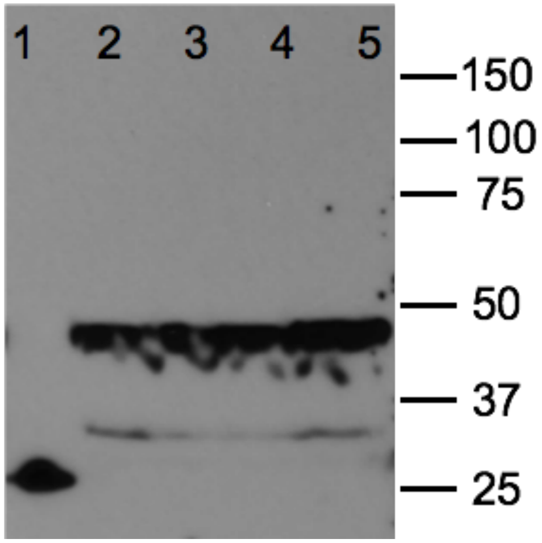

Supplementary Figure 15. Western blot analysis showing YFP-QueE expression. Western blot of cell lysates of strains expressing YFP-QueE from a plasmid. Lane 1 - cells expressing YFP only (SAM56); Lane 2 - (SAM144/ pSY76); Lane 3 - *ftsA-N170S* (SAM169/ pSY76); Lane 4 - *ftsZ-D234G* (SAM170/pSY76) and Lane 5 - *ftsA-R126C* (SAM171/ pSY76).

Supplementary Table 1. Cell length measurements

Strain[a]	Growth condition (in minimal medium)	Number of cells	Average cell length (μm)[b]	Histogram
Wild-type (TIM148)	+0.1 mM Mg^{2+}	500	3.2±0.8	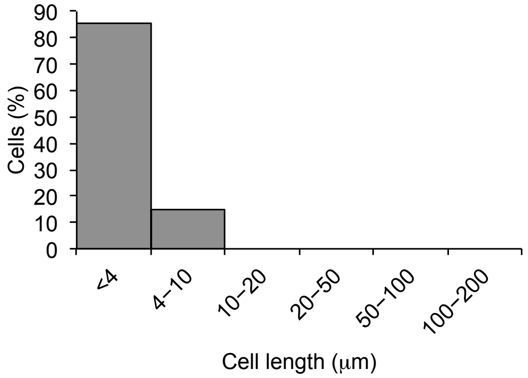
Wild-type (TIM148)	+0.1 mM Mg^{2+} +9μg/ml C18G	420	17.4±21.6[c]	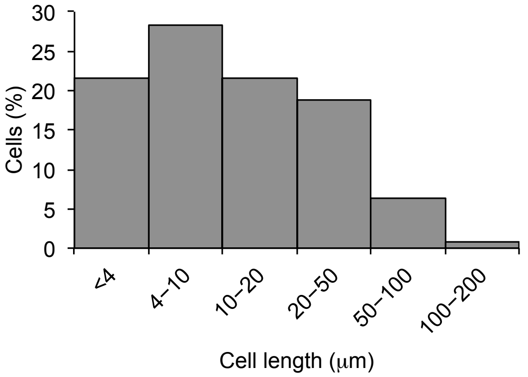
Wild-type (TIM92)	No added Mg^{2+}	500	3.0±1.0	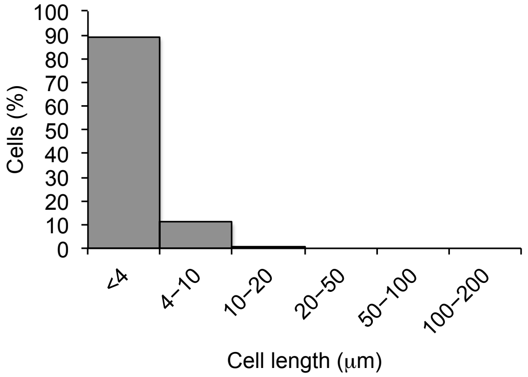

<table>
<tr><td></td><td></td><td></td><td></td><td></td></tr>
<tr><td>ΔmgrB
(AML20)</td><td>No added Mg^{2+}</td><td>262</td><td>24.3±26.8[c]</td><td>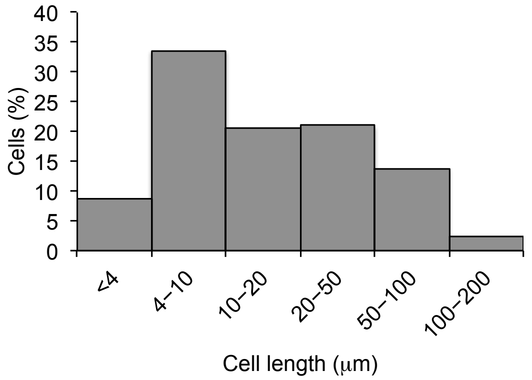
</td></tr>
<tr><td>ΔmgrB ΔqueE
(JNC21)</td><td>No added Mg^{2+}</td><td>449</td><td>3.7±1.7</td><td>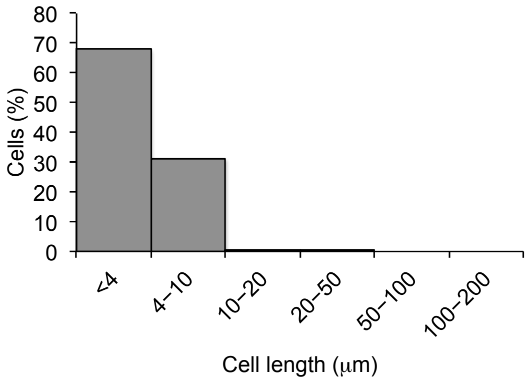
</td></tr>
<tr><td>ΔmgrB ΔyqcG
(SAM4)</td><td>No added Mg^{2+}</td><td>65</td><td>94.5±46.0[c]</td><td>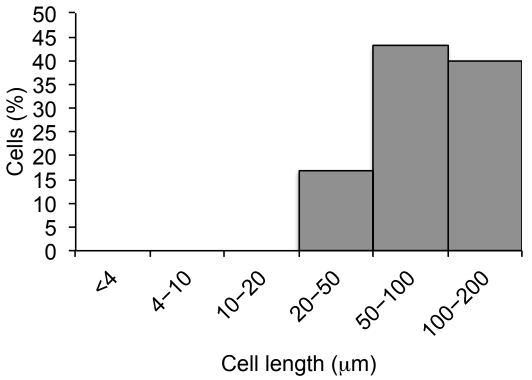
</td></tr>
<tr><td>ΔmgrB ΔyqcG
ftsA N170S
(AIC155)</td><td>No added Mg^{2+}</td><td>420</td><td>3.5±1.4</td><td>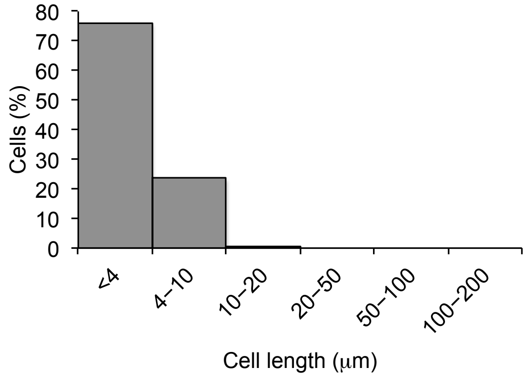
</td></tr>
</table>

ΔmgrB ΔyqcG ftsZ D234G (AIC156)	No added Mg^{2+}	247	4.6±2.7	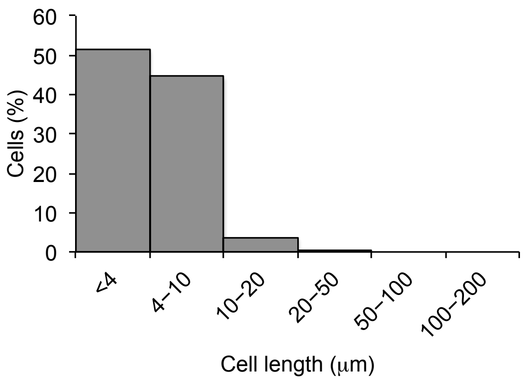
ΔmgrB ΔyqcG ftsA R126C (AIC157)	No added Mg^{2+}	338	4.6±3.1	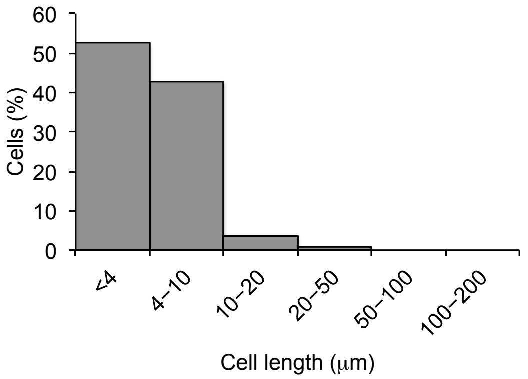

[a]See Table S2 for detailed information on strain genotypes.

[b]Mean length±standard deviation was calculated for the indicated number of cells (or filaments) using phase contrast images and ImageJ[3]/MicrobeJ plugin[4].

[c]Filaments >100 μm long are underrepresented because they frequently did not fit within the field of view of micrographs. For this reason, these averages are underestimates.

Supplementary Table 2. Strains and plasmids used in the study

Strain	Relevant genotype[a]	Reference/source
MG1655	λ[-] *rph-1*	*E. coli* Genetic Stock Center,

		CGSC no. 7740
BL21(DE3)	*E. coli* B F^- *dcm ompT hsdS* (r_B^- m_B^-) *gal* λ (DE3)	Novagen
PIR2	F-*Δlac*169 *rpo*S(am) *rob*A1 *cre*C510 *hsd*R514 *end*A *rec*A1 *uidA(ΔMluI)::pir*	Life Technologies
AIC155	MG1655 *ΔmgrB ΔyqcG ftsA-N170S* Kan^R λ_{att}::(P_{mgrB}-*yfp*) HK_{att}::(P_{tetA}-*cfp*)	This work
AIC156	MG1655 *ΔmgrB ΔyqcG ftsZ-D234G* Kan^R λ_{att}::(P_{mgrB}-*yfp*) HK_{att}::(P_{tetA}-*cfp*)	This work
AIC157	MG1655 *ΔmgrB ΔyqcG ftsA-R126C* Kan^R λ_{att}::(P_{mgrB}-*yfp*) HK_{att}::(P_{tetA}-*cfp*)	This work
AML17	MG1655 *ΔphoQ ΔmgrB::kan* λ_{att}::(P_{mgrB}-*yfp*) HK_{att} (P_{tetA}-cfp)	[5]
AML20	MG1655 *ΔmgrB* λ_{att}::(P_{mgrB}-*yfp*) HK_{att}::(P_{tetA}-*cfp*)	[5]
AML22	MG1655 *ΔmgrB::kan* λ_{att}::(P_{phoPQ}-*yfp*) HK_{att}::(P_{tetA}-*cfp*)	[5]
AMS3	MG1655 *ΔmgrB ΔsulA::kan* λ_{att}::(P_{mgrB}-yfp) HK_{att}::(P_{tetA}-cfp)	This work
EC448	MC4100 *Δ*(λ_{attL}-*lom*)::(*bla lacI*^{q} P_{208}-*ftsZ-gfp*)	[6]
GS0241	MG1655 *ΔyqcG::kan*	[7]
JNC19	MC4100 *ΔmgrB::kan Δ*(λ_{attL}-*lom*)::(*bla lacI*^{q}	This work

	P_{208}-*ftsZ-gfp*)	
JNC21	MG1655 *ΔmgrB ΔqueE::kan* λ_{att}::(P_{mgrB}-yfp) HK_{att}::(P_{tetA}-cfp)	This work
MMR160	MG1655 *ΔqueD ΔqueE::kan* λ_{att}::(P_{mgrB}-yfp) HK_{att}::(P_{tetA}-cfp)	This work
MMR161	MG1655 *ΔmgrB ΔqueE ΔqueD::kan* λ_{att}::(P_{mgrB}-*yfp*) HK_{att}::(P_{tetA}-*cfp*)	This work
RL1	MG1655 *ΔqueD::kan* λ_{att}::(P_{mgrB}-*yfp*) HK_{att}::(P_{tetA}-*cfp*)	This work
RL2	MG1655 *ΔmgrB ΔqueD::kan* λ_{att}::(P_{mgrB}-*yfp*) HK_{att}::(P_{tetA}-*cfp*)	This work
RL3	MG1655 *ΔqueC::kan* λ_{att}::(P_{mgrB}-*yfp*) HK_{att}::(P_{tetA}-*cfp*)	This work
RL4	MG1655 *ΔmgrB ΔqueC::kan* λ_{att}::(P_{mgrB}-*yfp*) HK_{att}::(P_{tetA}-*cfp*)	This work
SAM4	MG1655 *ΔmgrB ΔyqcG* λ_{att}::(P_{mgrB}-*yfp*) HK_{att}::(P_{tetA}-*cfp*)	This work
SAM51	MG1655 *ΔmgrB ΔyqcG*	This work
SAM54	MG1655 Φ(*queE*$^{+}$-*yfp*$^{+}$) Kan^{R}	This work
SAM55	MG1655 *ΔmgrB* Φ(*queE*$^{+}$-*yfp*$^{+}$) Kan^{R}	This work
SAM56	MG1655 *ΔmgrB ΔyqcG* Φ(*queE*$^{+}$-*yfp*$^{+}$) Kan^{R}	This work
SAM60	MG1655 *ΔphoQ* Φ(*queE*$^{+}$-*yfp*$^{+}$) Kan^{R}	This work

SAM96	MG1655 *ΔqueE*	This work
SAM141	MG1655 *ΔphoPphoQ* Φ(*queE*$^+$-*yfp*$^+$) Kan^R	This work
SAM142	MG1655 *ΔphoP* Φ(*queE*$^+$-*yfp*$^+$) Kan^R	This work
SAM144	MG1655 *ΔlacZYA ΔqueE::kan*	This work
SAM169	MG1655 *ΔmgrB ΔyqcG ftsA-N170S* Kan^R	This work
SAM170	MG1655 *ΔmgrB ΔyqcG ftsZ-D234G* Kan^R	This work
SAM171	MG1655 *ΔmgrB ΔyqcG ftsA-R126C* Kan^R	This work
TIM92	MG1655 λ_{att}::(P_{mgrB}-*yfp*) HK_{att}::(P_{tetA}-*cfp*)	[8]
TIM148	MG1655 λ_{att}::(P_{phoPQ}-*yfp*) HK_{att}::(P_{tetA}-*cfp*)	[8]
TIM210	MG1655 *ΔlacZYA* λ_{att}::(P_{mgrB}-*yfp*) HK_{att}::(P_{tetA}-*cfp*)	[1]
TIM229	MG1655 *ΔphoQ* λ_{att}::(P_{phoPQ}-*yfp*) HK_{att}::(P_{tetA}-*cfp*)	[8]
TIM233	MG1655 *ΔlacZYA ΔphoP* λ_{att}::(P_{mgrB}-*yfp*) HK_{att}::(P_{tetA}-*cfp*)	[8]
pAL8	pEB52 P_{trc}-*mgrB*, Amp^R	[5]
pBAD33	*ori*(p15A), P_{araBAD}-MCS *araC*, Cam^R	ATCC 87402
pBAD-FtsA*	pBAD33 *ftsA** (*ftsA R286W*), Cam^R	[9]
pCP20	*ori*(Ts) FLP-recombinase expression plasmid, Amp^R, Cam^R	[10]
pDSW914	*ftsP-gfp* under control of a weak IPTG inducible promoter, Amp^R	From D. S. Weiss

pEB52	pTrc99a with the NcoI site removed, Amp^R	Goulian lab collection
pEB112	pKD13 derivative with *yfpA206K-FRT-kan-FRT*, Amp^R	Goulian lab collection
pEG4	pBAD33 *ftsZ-mCherry*, Cam^R	[11]
pGB2	pSC101 derivative, Spc^R	[12]
pKD13	*oriR6K bla FRT-kan-FRT*, Amp^R	[10]
pLPQ*2	pGB2 *phoPphoQ*$_{chim}$, *phoQ*$_{chim}$ encodes a PhoQ chimera where the periplasmic domain of *E. coli* PhoQ is replaced with that of *P. aeruginosa*. Spc^R	[13]
pRL03	pEB52 P_{trc}-*queE*, Amp^R	This work
pRL27	*oriR6K*, Tn5-RL27, Kan^R	[14]
pSafA	pTrc99a *safA*, Amp^R	Goulian lab collection
pSY76	pEB52 *yfp-queE*, Amp^R	This work
pTM50	pET22b *phoP*-His_6, Amp^R	[8]
pTM69	pTrc99a *phoQ*, Amp^R	[15]
pTM153	pTrc99A *phoQ-T281R*, Amp^R	Goulian lab collection
pTrc99a	P_{trc}-MCS *lacI*^{q}, Amp^R	[16]

[a]Φ(*queE*$^+$-*yfp*$^+$) denotes an operon fusion of *queE* and *yfp*.

Amp^R, Cam^R, Kan^R, and Spc^R denote antibiotic resistance to ampicillin, chloramphenicol, kanamycin, and spectinomycin, respectively.

Supplementary Table 3. Primers used in the study

Name	Sequence (5' → 3')
Bs_queE-SacI-U1	GACTACGAGCTCAAAAGGGTGGTTTGAATGGCTAAAG
Bs_queE-BamHI-L1	AGTAGTGGATCCCTATTATACTCCGCGTTTGTTGCC
EMSA-lacZ-L1	TGTGCTGAATTCCGATTAAGTTGGGTAACG
EMSA-lacZ-U1	AAACCAGAATTCCGCCCAATACGCAAACCG
ftsA-U1	ACAGCAGCAGGCGCAAAC
ftsA-L1	TGTTCAACAGCATTACCGCCG
ftsZ-U1	AACGGTGAAGCTGAAGTAGAAAAAC
ftsZ-L1	CCGACACCCGTCGCCTGAAC
gfp-speI-l1	AATTAACTAGTTTTGTATAGTTCATCCATG
mgrB-prom-BamHI-L1	CGTGGATCCGTTTCACCTACCTTATGTCA
mgrB-prom-EcoRI-U1	GCGAATTCACCGTGCTGGTGCCTCTGGC
oriR6kseqprim1	GACACAGGAACACTTAACGGC
queE-BamHI-L1	CCCACACGGATCCGATTAGCCCTGGATGGGTAAAAT GGAGGAG

queE-pEB52-L1	CTTGCATGCCTGCAGGTCGACTCTA
queE-EcoRI-U1<br>pEB52	CCCACACGAATTCGTTGTGGCAGGATCTGCAAT
queE-prom-EcoRI-U1	CAGGAATTCATCTGGATAAATAAATTGAC
queE-prom-EcoRI-U2	GACGAATTCTATCCCATCATTACGTTTG
queE-prom-L1	AGCATTCTCTGTGAAGTGGATAATTG
queE-prom-L2	TGAGAAATTTATAATGCGCTATTTC
queE-yfp-lred-U1	CATAAATATCTAAATATTGCCtgaTTAAACATTTATAAG<br>CGTTATAAATGGGCCGGGTACCTAGAATTAAAGAGG
queE-yfp-lred-L1	GGTAAAATGGAGGAGTTTTCAGAGGCGACAAACAATA<br>TAAATGAGTAGAAGCTTGAGCGATTGTGTAGGCTGG
SacI-RBS-YFP-u1	CCGGGTGCCGAGCTCAAAGAGGAGAAATTAAGCATG<br>CGTAAAGGAGAAGAACT
tnmodRkan1	CTCCCTCACTTTCTGGCTGG
xbaI-queE-U2	TACTCTAGAATGCAGTACCCGATTAACGAGATG
ygcF_L_2902682	TGGAGGAGTTTTCAGAGGCGACA
ygcF_R_2903498	ATGATTGTTGTGGCAGGATCTGCA

Supplementary References

1. Miyashiro, T. & Goulian, M. High stimulus unmasks positive feedback in an autoregulated bacterial signaling circuit. *Proceedings of the National Academy of Sciences of the United States of America* **105**, 17457-17462 (2008).
2. McCarty, R.M. & Bandarian, V. Biosynthesis of pyrrolopyrimidines. *Bioorg Chem* **43**, 15-25 (2012).
3. Schneider, C.A., Rasband, W.S. & Eliceiri, K.W. NIH Image to ImageJ: 25 years of image analysis. *Nat Methods* **9**, 671-5 (2012).
4. Jiang, C., Brown, P.J., Ducret, A. & Brun, Y.V. Sequential evolution of bacterial morphology by co-option of a developmental regulator. *Nature* **506**, 489-93 (2014).
5. Lippa, A.M. & Goulian, M. Feedback inhibition in the PhoQ/PhoP signaling system by a membrane peptide. *PLoS genetics* **5**, e1000788 (2009).
6. Weiss, D.S., Chen, J.C., Ghigo, J.M., Boyd, D. & Beckwith, J. Localization of FtsI (PBP3) to the septal ring requires its membrane anchor, the Z ring, FtsA, FtsQ, and FtsL. *J Bacteriol* **181**, 508-20 (1999).
7. Hobbs, E.C., Astarita, J.L. & Storz, G. Small RNAs and small proteins involved in resistance to cell envelope stress and acid shock in Escherichia coli: analysis of a bar-coded mutant collection. *Journal of bacteriology* **192**, 59-67 (2010).
8. Miyashiro, T. & Goulian, M. Stimulus-dependent differential regulation in the Escherichia coli PhoQ PhoP system. *Proc Natl Acad Sci U S A* **104**, 16305-10 (2007).

9. Geissler, B., Elraheb, D. & Margolin, W. A gain-of-function mutation in ftsA bypasses the requirement for the essential cell division gene zipA in Escherichia coli. *Proceedings of the National Academy of Sciences of the United States of America* **100**, 4197-4202 (2003).

10. Datsenko, K.A. & Wanner, B.L. One-step inactivation of chromosomal genes in Escherichia coli K-12 using PCR products. *Proceedings of the National Academy of Sciences of the United States of America* **97**, 6640-6645 (2000).

11. Galli, E. & Gerdes, K. Spatial resolution of two bacterial cell division proteins: ZapA recruits ZapB to the inner face of the Z-ring. *Mol Microbiol* **76**, 1514-26 (2010).

12. Churchward, G., Belin, D. & Nagamine, Y. A pSC101-derived plasmid which shows no sequence homology to other commonly used cloning vectors. *Gene* **31**, 165-71 (1984).

13. Lesley, J.A. & Waldburger, C.D. Comparison of the Pseudomonas aeruginosa and Escherichia coli PhoQ sensor domains: evidence for distinct mechanisms of signal detection. *J Biol Chem* **276**, 30827-33 (2001).

14. Larsen, R.A., Wilson, M.M., Guss, A.M. & Metcalf, W.W. Genetic analysis of pigment biosynthesis in Xanthobacter autotrophicus Py2 using a new, highly efficient transposon mutagenesis system that is functional in a wide variety of bacteria. *Archives of microbiology* **178**, 193-201 (2002).

15. Goldberg, S.D., Soto, C.S., Waldburger, C.D. & Degrado, W.F. Determination of the physiological dimer interface of the PhoQ sensor domain. *Journal of molecular biology* **379**, 656-665 (2008).
16. Amann, E., Ochs, B. & Abel, K.J. Tightly regulated tac promoter vectors useful for the expression of unfused and fused proteins in Escherichia coli. *Gene* **69**, 301-15 (1988).
